# A dimeric holin/antiholin complex controls lysis by phage T4

**DOI:** 10.3389/fmicb.2024.1419106

**Published:** 2024-09-05

**Authors:** Jan Michel Frederik Schwarzkopf, Denise Mehner-Breitfeld, Thomas Brüser

**Affiliations:** Institute of Microbiology, Leibniz Universität Hannover, Hanover, Germany

**Keywords:** protein–protein interaction, membrane protein, bacteriophage, holin, antiholin, phage T4, lysis inhibition, *Escherichia coli*

## Abstract

Lytic phages control the timepoint of host cell lysis by timing the holin-mediated release of cell wall-degrading endolysins. In phage T4, the antiholin RI inhibits the holin T, thereby preventing the early release of the T4 endolysin and lysis. The antiholin achieves lysis inhibition (LIN) in response to phage superinfections, thereby increasing the chance for lysis in an environment with a lower phage concentration. The holin T consists of a small N-terminal cytoplasmic domain, a transmembrane helix, and a periplasmic C-terminal domain. The antiholin is targeted to the periplasm by a cleavable signal peptide. Recently, the periplasmic soluble domains of the holin and the antiholin were found to form T_2_/RI_2_ tetramers in crystals. To investigate the functional relevance of this complex, we reconstituted LIN in a phage-free system, using only RI, T, and endolysin, and combined targeted mutagenesis with functional analyses. Inactivation of the RI signal peptide cleavage site did not abolish LIN, indicating that RI can function in a membrane-bound state, which argued against the tetramer. This led to analyses showing that only one of the two T/RI interfaces in the tetramer is physiologically relevant, which is also the only interaction site predicted by AlphaFold2. Some holin mutations at this interaction site prevented lysis, suggesting that the RI interaction likely acts by blocking the holin oligomerization required for hole formation. We conclude that LIN is mediated by a dimeric T/RI complex that, unlike the tetramer, can be easily formed when both partners are membrane-anchored.

## 1 Introduction

Lytic phages tightly regulate the lysis of their host cells by controlling the access of phage-encoded muralytic endolysins to the bacterial cell wall (Young, 2014). Endolysins can be released via holes formed by membrane proteins that are termed canonical holins (Young, 1992). The regulation of lysis timing occurs by controlling the activity of the holins (Catalão et al., 2013). The timing of lysis can be achieved by the regulated formation of holins and their accumulation in the cytoplasmic membrane until a critical density is reached for hole formation (White et al., 2011). Many holins are regulated by specific antiholins that sense superinfections and delay lysis (Abedon, 2019). T4 is the prototype for studies on this lysis inhibition (LIN), in which two antiholins, RI and RIII, are involved in binding the periplasmic and cytoplasmic domains of the holin T, respectively (Ramanculov and Young, 2001a; Chen and Young, 2016). The cytoplasmic RIII alone cannot establish a stable LIN and stabilizes the inhibitory effect of the periplasmic RI, which is the LIN master regulator (Chen and Young, 2016). RIII also has additional regulatory functions related to its interaction with the ribosomal protein S1 (Juškauskas et al., 2022). Recently, we demonstrated that, in contrast to previous reports, RI has a cleavable Sec signal peptide (Mehner-Breitfeld et al., 2021). This simplified the current view of phage lysis regulation and implied a fundamentally different interpretation of a recently published tetrameric T2/RI2 holin–antiholin complex that has been crystallized after mixing the periplasmic domains of the holin T and the antiholin RI (Krieger et al., 2020). As the N-termini of the holins and antiholins in the crystallized tetramer point in opposite directions, it was difficult to explain the membrane anchoring of all four subunits of the complex, and the finding of RI signal peptide cleavage permitted a simplified revised model in which only the holins are membrane-anchored (Mehner-Breitfeld et al., 2021). However, this postulated new model assumed that only the mature RI that lacks its membrane anchoring signal peptide could form the inhibitory complex.

This study addresses the structural model for the inhibitory T/RI interaction experimentally. In contrast to expectations, we found that the antiholin RI inhibits lysis in a non-processed membrane-anchored state. Signal peptide cleavage is slow, and sufficient precursor is present in the membrane to enable the inhibitory function. Further analyses indicated that the previously crystallized tetrameric T2/RI2 inhibitory complex is most likely crystallization-induced and that a dimeric structure is of physiological relevance, which explains how the two proteins can interact in a membrane-bound state.

## 2 Materials and methods

### 2.1 Strains and growth conditions

*Escherichia coli* strain ER2566 (NEB, Ipswich, United States) was used for heterologous protein production, while *E. coli* XL1-Blue Mrf’ Tet (Stratagene, La Jolla, CA, United States) or DH5α *λ pir*^+^ were used for cloning. Cells were grown aerobically in the LB medium (1% tryptone, 0.5% yeast extract, and 0.5% NaCl) at 37°C with appropriate antibiotics (100 μg/mL of ampicillin, 25 μg/mL of chloramphenicol, and 50 μg/mL of kanamycin). A measure of 0.1% rhamnose was used to induce P*_rhaB_*-dependent gene expression at indicated time points. A measure of 1% glucose was added to overnight cultures for the repression of gene expression. For growth assays, flasks with 25 mL of LB medium supplemented with 0.01% glycerol were inoculated to an OD_600_ of 0.1 from an overnight culture, and cultures were grown aerobically. In experiments with translation inhibition, 1 L cultures were treated with 25 μg/mL chloramphenicol, and samples equivalent to 50 mL of OD_600_ = 1 were taken at indicated time points and analyzed.

### 2.2 Plasmids and genetic methods

T was N-terminally fused to a strep-SUMO-tag. All vectors were cloned via standard methods. Primers are listed in Table 1. pBW-*t*-H6 was constructed by amplifying *t* from the T4 genome using the primer *t*-NdeI-F in combination with the primer *t*-BamHI-R2, and cloning *t* into pBW-*tatA*-H6 (Berthelmann et al., 2008). To construct pCOLA-*strep-sumo-t*, *strep-sumo-t* was amplified with pBW-*t*-H6 as a template, using the primers *str-su-t*-NcoI-F and *str-su-t*-HindIII-R. The fragment was cloned into the corresponding sites of pCOLA-*rI*-HA (Mehner-Breitfeld et al., 2021). The gene *rI* was rhamnose-inducibly expressed from pBW22-based vectors (Wilms et al., 2001) to produce RI with a C-terminally fused hexahistidine-tag. pBW-*rI*-H6 was constructed analogously to pBW-*t*-H6, after amplifying *rI* from T4 genome using the primers *rI*-NdeI-F and *rI*-BamHI-R2. The T4 endolysin-encoding gene *e* was expressed constitutively from the vector pLysS-t4l after exchanging the T7 lysozyme-encoding gene in pLysS with the cysteine-free variant of *e*, which was a gift of Gerd Bange (Marburg), using the primers *t4l*-NdeI-F and *t4l*-XhoI-R. A S42A mutation in *e* encoded on pBAD-H10-T4L-ycdB(ss) was reversed by a QuikChange^™^ mutagenesis (Stratagene) using the forward primer *t4l*-A42S-F in conjunction with the reverse primer that covers the identical sequence region. To insert *e* into pLysS, the vector was amplified using the primers pLySlin-NdeI-R and pLysSlin-XhoI. Single amino acid exchanges in RI or T were introduced by QuikChange mutagenesis (Stratagene) of pBW-*rI*-H6 or pCOLA-*strep-sumo-t*, using the primers listed in Table 1.

**Table 1:** Primers used in this study.

Standard primers	SEQUENCE (5′ > 3′)
t-NdeI-F	ATGTACATATGGCAGCACCTAGAATATC
t-BamHI-R2	ATGTAGGATCCTTTAGCCCTTCCTAATATTCTG
str-su-t-NcoI-F	ATATACCATGGATGGGTTGGAGCCACCCG
str-su-t-HindIII-R	ACGCGAAGCTTTTATTTAGCCCTTCCTAATATTCTGG
rI-NdeI-F	ATGTACATATGGCCTTAAAAGCAACAG
rI-BamHI-R2	ATGTAGGATCCTTCAGTCTCCAATTTAATGTTCATA
t4l-NdeI-F	GCGCGCATATGAATATATTTGAAATGTTACGTATAG
t4l-XhoI-R	CGCGTATAAAAATCTAGGATCCTAACTCGAG
pLySlin-NdeI-R	GCGCCATATGTTTCCTCCTTTCCTTTTTAATC
pLysSlin-XhoI	ATATACTCGAGGAACTCACTAAAGGGAGACC

QuikChange primers^a^	SEQUENCE (5′ > 3′)^a^
rI-A24P-F	GTTTTATCTCCATCGATTGAACCGAATGTCGATCCTCATTTTG
rI-N25A-F	CCATCGATTGAAGCGGCAGTCGATCCTCATTTTG
rI-F30A-F	GTCGATCCTCATGCCGATAAATTTATG
rI-D31A-F	GAAGCGAATGTCGATCCTCATTTTGCTAAATTTATGGAATCTGGTATTAG
rI-D31N-F	CGATTGAAGCGAATGTCGATCCTCATTTTAATAAATTTATGGAATCTGGTATTAGGCAC
rI-M34A-F	CATTTTGATAAATTTGCGGAATCTGGTATTAG
rI-V41A-F	GTATTAGGCACGCCTATATGCTTTTTG
rI-V41G-F	GTATTAGGCACGGCTATATGCTTTTTG
rI-Y42A-F	GAATCTGGTATTAGGCACGTTGCAATGCTTTTTGAAAATAAAAG
rI-Y42L-F	GAATCTGGTATTAGGCACGTTCTCATGCTTTTTGAAAATAAAAGC
rI-Y42F-F	GGTATTAGGCACGTTTTTATGCTTTTTG
rI-S53A-F	GAAAATAAAAGCGTAGAATCGGCCGAACAATTCTATAGTTTTATG
rI-S53A, E54A-F	GTAGAATCGGCCGCACAATTCTATAG
rI-E54A-F	GTAGAATCGTCTGCACAATTCTATAG
rI-F56A-F	CGTAGAATCGTCTGAACAAGCGTATAGTTTTATGAGAACG
rI-F56L-F	GCGTAGAATCGTCTGAACAACTGTATAGTTTTATGAGAACGACC
rI-Y57A-F	CTGAACAATTCGCTAGTTTTATGAG
rI-R61A-F	CAATTCTATAGTTTTATGGCAACGACCTATAAAAATGAC
rI-R61Q-F	CAATTCTATAGTTTTATGCAGACGACCTATAAAAATGAC
rI-K65A-F	GAACGACCTATGCAAATGACCCGTG
rI-K65L-F	GAACGACCTATCTAAATGACCCGTG
rI-G79A-F	GTATAGAGCGAGCAGCGGAGATGGC
rI-Y86A-F	GATGGCACAATCAGCCGCTAGAATTATG
rI-Y86L-F	GATGGCACAATCACTCGCTAGAATTATG
t-R104A-F	CTTTAGTGCGGTGTATTCTTTCGCCCCTAAAAACTTAAACTATTTTG
t-Y110A-F	CCCTAAAAACTTAAACGCCTTTGTTGATATTATAG
t-F111A-F	CCTAAAAACTTAAACTATGCCGTTGATATTATAGCATAC
t4l-A42S-F	CATCACTTAATGCTAGCAAATCTGAATTAG

a Corresponding reverse primers covered the same sequence.

### 2.3 Biochemical methods

For subcellular fractionation in periplasm/membrane/cytoplasm fractions, 50 mL of exponentially growing cultures were sedimented (3,260 × g, 4°C), and cells were resuspended in 20 mL of 20% sucrose/10 mM Tris-HCl, pH 8.0/1 mM EDTA, followed by incubation for 10 min at room temperature. The cells were again sedimented (4,500 × g, 4°C), and the supernatant-free cell pellet was resuspended in ice-cold 1 mL of 5 mM MgSO_4_ and incubated for 20 min on ice. After this osmotic shock, the cells were sedimented (9,500 × g, 4°C), and the periplasm (supernatant) was carefully collected. The cell pellet was then resuspended in 1 mL of 5 mM MgSO_4_, and the cells were disintegrated by sonification (3 × 30 s with 30 s interruptions) on ice, followed by sedimentation of cell debris (20,000 × g, 10 min, 4°C), and ultracentrifugation (130,000 × g, 30 min, 4°C) for the separation of membranes and cytoplasmic proteins. For the generation of whole cell extracts, cells from 50 mL of cultures of OD_600_ = 1 were harvested by centrifugation (3,260 × g, 4°C), resuspended in 1 mL of 50 mM Tris–HCl pH 8.0, and disrupted by sonification. For the preparation of soluble and membrane fractions, this extract was subjected to ultracentrifugation (130,000 × g, 30 min, 4°C).

SDS-PAGE was carried out according to the standard protocol of Lämmli, using 12.5% T gels for the analyses of the holin T and the YidC control, and 15% T gels for all other analyses (Laemmli, 1970). Western blotting was carried out using standard procedures (Towbin et al., 1979). Blots were developed using polyclonal antibodies directed against the hexahistidine Tag (Qiagen), the T4 lysozyme (Millipore), or YidC (gift of Andreas Kuhn, Hohenheim), or monoclonal mouse antibodies directed against beta-lactamase (Acris). Horseradish peroxidase (HRP)-conjugated goat anti-rabbit (Roth, Karlsruhe, Germany) or goat anti-mouse antibodies served as secondary antibodies, respectively. Strep-SUMO-T and biotin carboxyl carrier protein (BCCP) were detected using HRP-coupled Strep-Tactin (IBA Lifesciences GmbH, Göttingen, Germany). The ECL system was used for detection (GE Healthcare).

### 2.4 Computational methods

Protein structures and complexes were predicted using ColabFold v1.5.5/AlphaFold2 (Mirdita et al., 2022) under default settings. Homologs were identified using BLASTP (Altschul et al., 1990), alignments were conducted using Clustal Omega (Sievers et al., 2011), and the conservation of residues at the sequence positions was analyzed using WebLogo (Crooks et al., 2004).

## 3 Results

### 3.1 Membrane-anchored antiholin RI inhibits the holin T in a reconstituted LIN system

**Figure 1:**
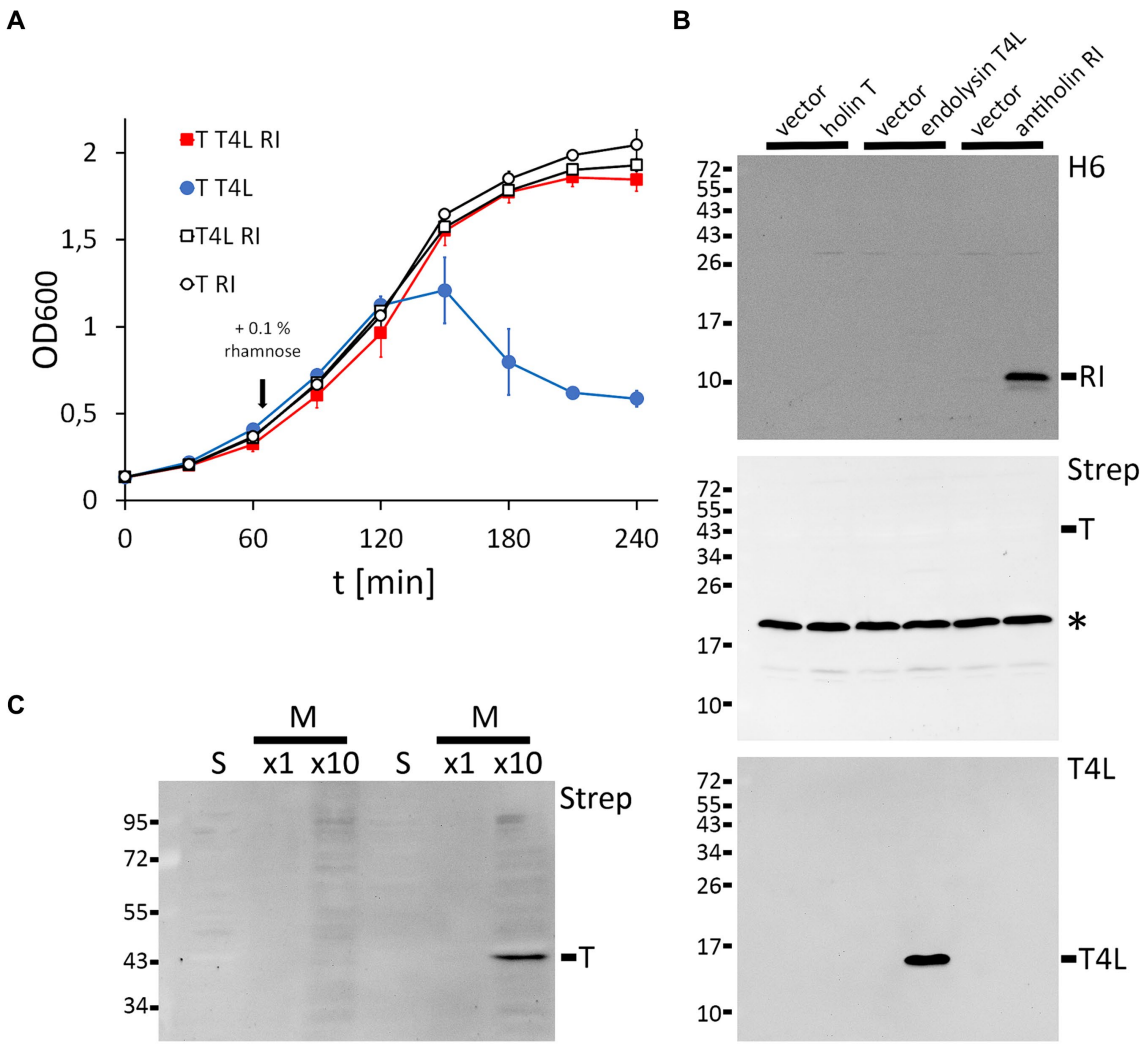
Reconstitution of lysis inhibition (LIN). **(A)** Growth curves of *E. coli* strains producing the indicated combinations of holin (T), T4 lysozyme (T4L), and antiholin (RI). Antiholin production was induced by the addition of 0.1% rhamnose at the indicated time point, whereas holin T and T4L were constitutively produced. Note that RI inhibits lysis (compare the red and blue curves), and no lysis is observed in the absence of the holin or the T4 lysozyme. **(B)** Western blots confirming the production of C-terminally hexahistidine-tagged RI (upper blot) and T4 lysozyme (lower blot), as produced by the individual expression systems. Empty vectors were used as controls. N-terminally Strep-SUMO-tagged T as produced by the leakage of the non-induced T7 promoter system was below the detection limit (middle blot). The asterisks indicate the position of the biotin carboxyl carrier protein (BCCP). **(C)** Detection of the holin required induction of gene expression by 1 mM IPTG and a 10-fold concentration of the proteins. Western blot of soluble (s) and membrane (m) fractions after subcellular fractionation.

Thus far, the function of several individual positions in the holin T and antiholin RI of phage T4 has been investigated using either complete phages or vector-based expression of the lambda lysis cassette in which the lambda holin gene was exchanged by the T4 holin *t* gene in combination with a second vector for *rI* expression (e.g., Ramanculov and Young, 2001b; Tran et al., 2007). To examine the functional role of potentially important and so far unstudied specific RI or T amino acids in a less complex system without lambda genes, we reconstituted LIN in *Escherichia coli* using only the three T4 components: holin T, antiholin RI, and T4 lysozyme. We certainly did not intend to question any results obtained previously using the lambda cassette system. After the optimization of the expression systems for each of the components, we were successful with a non-induced pCOLA-ori/T7 promoter combination for the holin gene (pCOLA-*strep-sumo-t*), a rhamnose-inducible pMB1-ori/P*_rhaB_*-promoter system for the antiholin gene (pBW-*rI*-H6), and constitutive p15A-ori/P_tet_-promoter system for the T4 lysozyme gene (pLysS-*t4l*). Using these three compatible plasmids and an LB medium that was supplemented with 0.01% glycerol, we achieved LIN by rhamnose-induced production of the antiholin RI (Figure 1A). As expected, lysis depended on the holin T and the T4 lysozyme. The holin T was produced with an N-terminal Strep-SUMO tag, which facilitated detection without compromising its function. Western blots confirmed RI and T4L production with the individual expression systems, whereas holin T was functional with an abundance below detection limit (Figure 1B). For the detection of the holin T, the holin gene had to be induced and the membrane fraction had to be 10-fold concentrated (Figure 1C). This result suggests that already very low-abundant holin can catalyze the release of endolysins into the periplasm.

**Figure 2:**
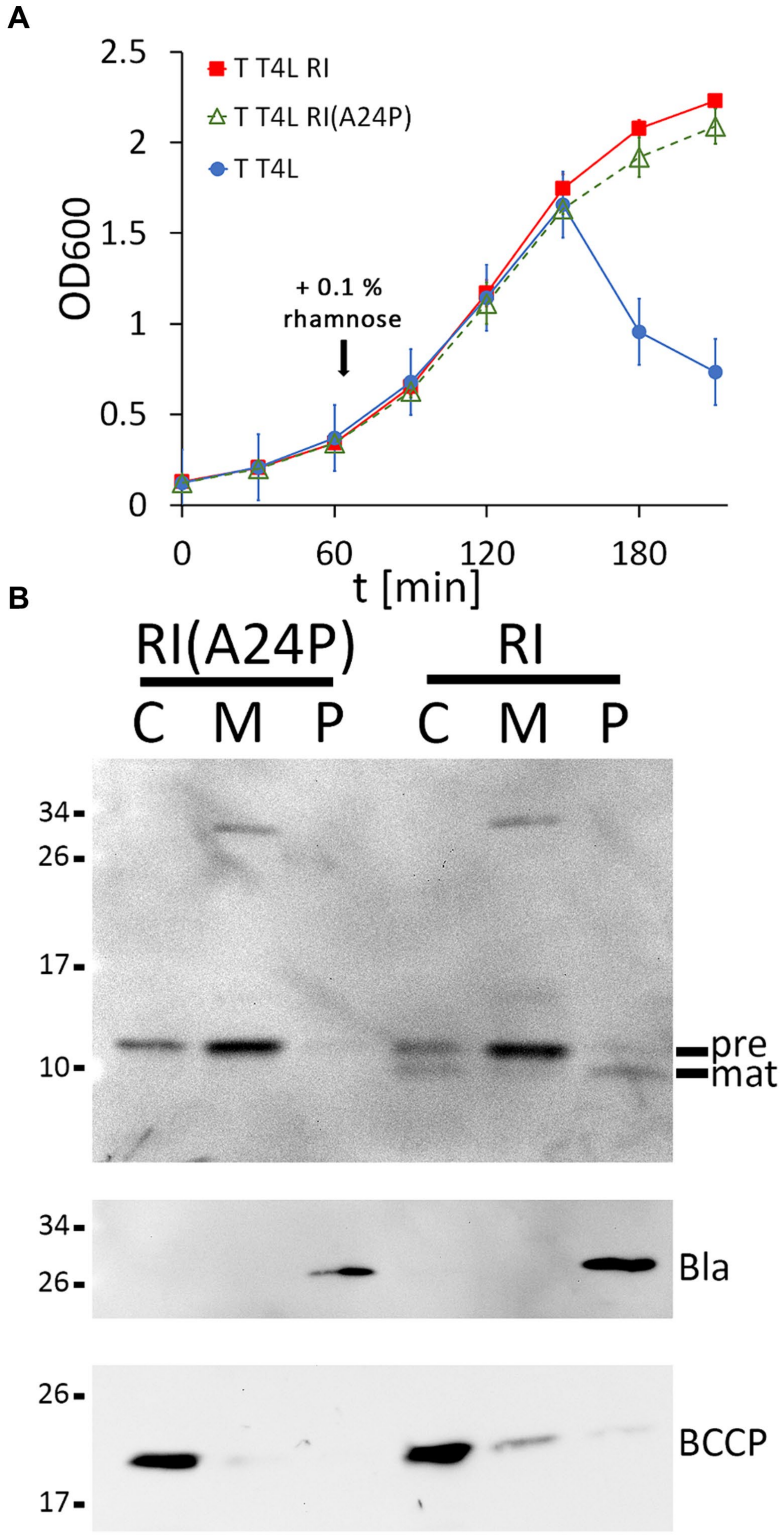
Membrane-bound RI(A24P) inhibits T. **(A)** Growth curves demonstrating lysis inhibition (LIN) as observed with RI(A24P) (green curve). As positive and negative controls, strains with the functional LIN system and the lysis system without RI were examined in parallel (red and blue curves). **(B)** Detection of the RI(A24P) variant and of wild-type RI in subcellular fractions. SDS-PAGE/Western blot analysis using antibodies recognizing the C-terminal hexahistidine-tag. Pre, precursor of RI; mat, processed mature RI, lacking the signal peptide. Note that the signal peptide is only processed in wild-type RI, whereas the A24P mutation abolished cleavage and release of mature RI into the periplasm. Fractionation controls are shown in the two blots below, including the detection of cytoplasmic BCCP and periplasmic β-lactamase (Bla).

Once the functional LIN system was reconstituted, we went one step further and used this system to examine whether cleavage of the RI signal peptide is important for LIN. To approach this, we generated an A24P exchange in the signal peptide cleavage site, which already has been shown to abolish cleavage (Mehner-Breitfeld et al., 2021), and we tested the potential effects of this exchange on LIN (Figure 2A). Wild-type RI was included as a control in the analysis. To our surprise, LIN did not depend on signal peptide cleavage. When we examined the presence of RI precursor and signal-peptide-cleaved mature RI in subcellular fractions, the processing of RI was found to be completely abolished by the A24P mutation, resulting in membrane-bound precursor and some precursor in the cytoplasmic fraction. We detected a partial signal peptide cleavage to mature size in the cytoplasmic fraction, which also was abolished by the A24P exchange, indicating that signal peptide cleavage continued after osmotic shock, resulting in the contamination of the cytoplasmic fraction with mature RI continuously released from the membranes. As in the case of the wild-type RI, we observed abundant precursor of RI(A24P) in the membrane fraction. Only a rather small portion of the wild-type RI was processed and released into the periplasm in this expression system, indicating that signal peptide cleavage-mediated release into the periplasm was slow. The slow processing of wild-type RI and the functionality of the non-processed RI(A24P) precursor suggested that low-efficient processing could be a desired characteristic of the RI antiholin, which inhibits the holin in a membrane-anchored state (Figure 2B).

### 3.2 Only one of the two T/RI interaction regions of the T2/RI2 structure is relevant for LIN, suggesting a dimeric T/RI complex structure

**Figure 3:**
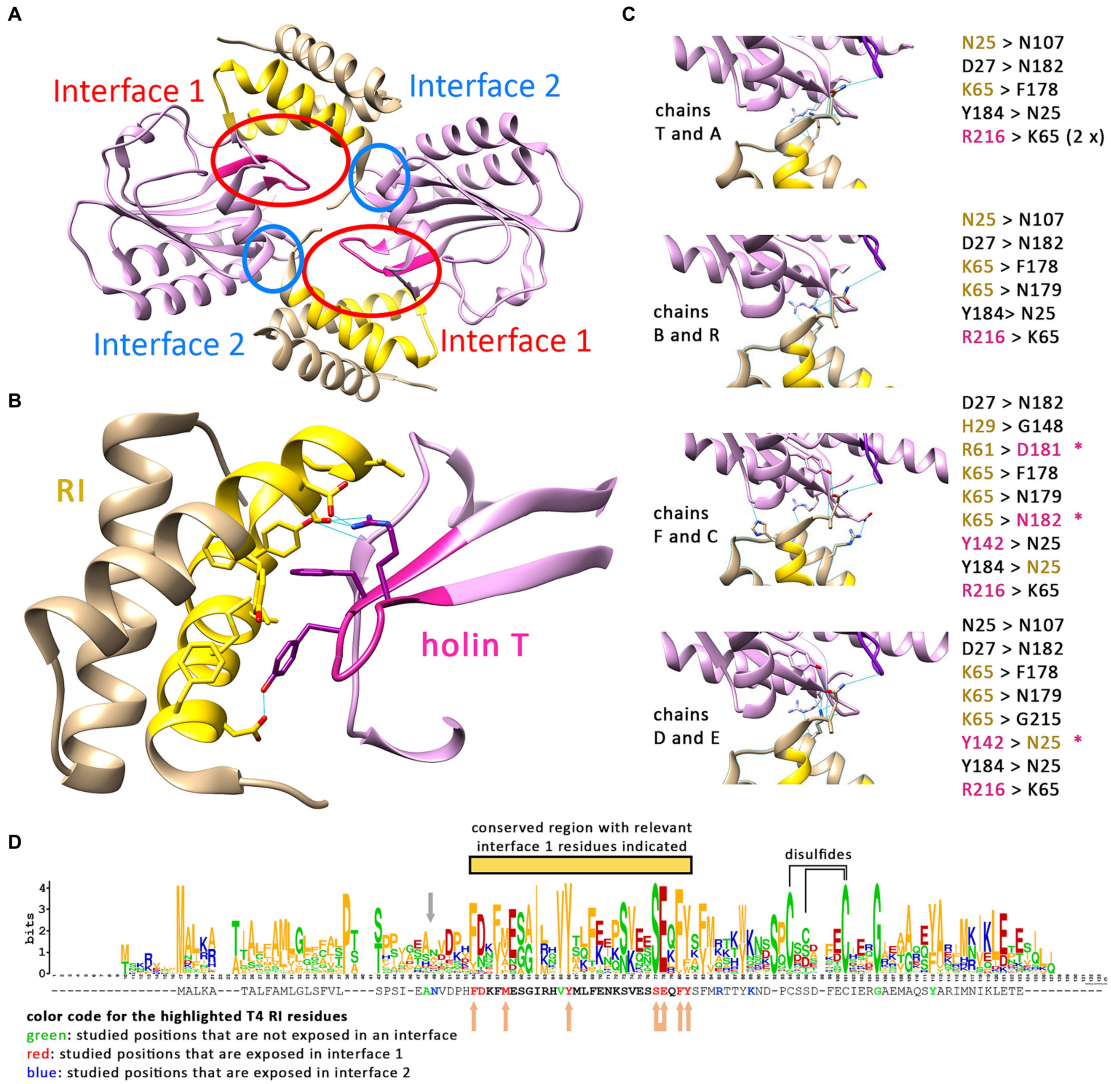
Only one interface of the crystallized T/RI tetramer is conserved. **(A)** The crystallized T/RI tetramer contains two different interfaces between holin T (purple) and antiholin RI (beige) that occur twice. **(B)** Interface 1 comprises highly conserved regions of RI (gold) and T (magenta), and several amino acid residues (shown) were proven important for LIN or lysis function. Predicted hydrogen bonds (indicated as blue lines) play only a minor role in interface 1. **(C)** Interface 2 is smaller, and interactions differ in the two tetramers of the asymmetric unit in structure PDB 6PXE. The chains are indicated at the left of the respective interfaces, with predicted hydrogen bonds as blue lines, and the hydrogen bond donors and acceptors are listed on the right. Black residues contribute only to backbone positions for hydrogen bonding. Beige and magenta residues are RI or T residues, respectively, that form hydrogen bonds with a side chain atom. The asterisks indicate hydrogen bonding between the side chains. **(D)** WebLogo analysis of a Clustal Omega alignment of 168 RI sequences (alignment shown in Supplementary Table S1). The T4 RI sequence from this alignment is indicated below the WebLogo output, with mutated amino acid positions in interfaces 1 and 2 highlighted in red and blue, respectively. All residues of interface 1 are located in a highly conserved region (yellow bar). Studied positions that are not exposed in an interface are highlighted in green. The gray arrow indicates the suboptimal signal peptidase cleavage site. Beige arrows indicate positions in interface 1 at which mutations caused LIN deficiency. In the case of the connected arrow, only the double mutation caused LIN deficiency. Cysteines that likely form disulfides, according to studies on T4 RI (Moussa et al., 2012), are connected by a line above the WebLogo output.

It is currently believed that a T_2_:RI_2_ tetramer, as crystallized from mixtures of purified soluble domains of T and RI, is the structural basis for LIN (Krieger et al., 2020). In the tetramer, the N-termini of the crystallized soluble domains point to divergent directions, and it therefore was difficult to explain how the four transmembrane domains that were all missing in the structure could reach the membrane. When it was recognized that RI possesses an N-terminal cleavable signal peptide, only the two remaining transmembrane domains of the holin T needed to face the membrane, which changed the structural model (Mehner-Breitfeld et al., 2021). However, as we now found that the membrane-anchored antiholin RI is interacting with the holin, resulting in LIN (Figure 2), also the revised tetramer model with only two membrane anchors could not be correct. We thus had a closer look at the relevance of the holin–antiholin interactions that were seen with the tetrameric structure of the soluble domains. In the crystallized T/RI tetramer, each subunit interacts with two neighboring subunits, and consequently, there are two distinct T–RI interfaces (Figure 3A). We reasoned that if only one of the two interfaces is relevant, then the tetramer could represent a crystallization-induced dimer of dimers, and the holin and antiholin could both be easily membrane-anchored in dimers.

The two interfaces differ in size, in the mode of interaction, and in the conservation of interacting residues among T4 family phages (Figures 3B–D). The larger interface (now termed interface 1) comprises several aromatic and aliphatic residues, and interaction therefore is likely driven by the hydrophobic effect (Figure 3B). To address the relevance of these interface 1 interactions, we generated RI variants carrying single exchanges of potentially relevant residues and examined their impact on LIN (Table 2 and Supplementary Figure S1). The Y42A, Y42L, and F56A variants abolished LIN. In the case of the Y42 exchanges, we wanted to address whether the Y42 side chain hydroxyl group is important or whether its aromatic properties are required for LIN. We therefore also analyzed a Y42F variant, and while Y42A and Y42L were non-functional, the Y42F exchange did support LIN, demonstrating that the aromaticity at this position is important. We also found that F30A, M34A, and Y57A mutations of the antiholin reduced the LIN, further supporting the view that the hydrophobic effect triggers the interaction. Interestingly, the holin variants Y110A and F111A strongly affected the lysis function of the holin, thus not permitting an analysis of LIN. As these residues are part of interaction surface 1, these observations suggest that RI binding to the holin likely blocks directly the holin multimerization site, which would be a reasonable and simple mechanism of inhibition. We also considered the potential relevance of the few predicted hydrogen bonds for the interaction at interface 1, and we generated RI variants S53A and E54A to address this question. Both residues form hydrogen bonds to R104 in the holin T. However, while the removal of individual hydrogen bonds had no effect, the combination of both mutations abolished LIN functionality. This suggests that hydrogen bonds can contribute to the interaction (Table 2). When we analyzed an R104A variant of the holin, we found a reduction in LIN, which agrees with the view that hydrogen bonding plays some role, but LIN was not abolished and thus hydrogen bonding is not the key factor for the interaction. In summary, our data show that interface 1 is important for LIN, and hydrophobic or aromatic interactions of RI positions F30, M34, Y42, F56, and Y57 are of key importance in that interaction.

**Table 2:** Effects of mutations in the antiholin RI and in holin T on LIN functionality.

RI analyses			
Variants	Region	Conservation	LIN defect
F30A	Interface 1	yes	strongly reduced
D31A	Interface 1	yes	no
D31N	Interface 1	yes	no
M34A	Interface 1	yes	reduced
Y42A	Interface 1	yes	yes
Y42L	Interface 1	yes	yes
Y42F	Interface 1	yes	no
S53A	Interface 1	yes	no
E54A	Interface 1	yes	no
S53A E54A	Interface 1	yes	yes
F56A	Interface 1	yes	yes
F56L	Interface 1	yes	no
Y57A	Interface 1	yes	reduced
N25A	Interface 2	no	no
R61A	Interface 2	no	no
R61Q	Interface 2	no	no
N25A-R61A	Interface 2	no	no
K65A	Interface 2	no	no
K65L	Interface 2	no	no
A24P	Signal cleavage site	yes	no
V41A	control	yes	no
V41G	control	yes	no
G79A	control	yes	no
Y86A	control	yes	no
Y86L	control	yes	no

T analyses			
Variants	Conservation	Supports lysis	LIN defect
R104A	yes	yes	reduced
Y110A	yes	reduced	n.d.^a^
F111A	yes	no	n.d.^a^

a n.d., not detectable; cannot be analyzed because mutation causes lysis defect.

The smaller interface 2 has no hydrophobic character, and interactions are mainly mediated by hydrogen bonds. A closer analysis of the interface 2 interactions in the two tetramers of the asymmetric unit in structure PDB 6PXE (Krieger et al., 2020) revealed that the hydrogen bonding at the small interface 2 differs significantly in the four cases (Figure 3C). Notably, most hydrogen bonding involves backbone amide hydrogens and carbonyl oxygens, indicating that specific side chains are not involved in these cases, which is unexpected for a physiologically relevant interaction. However, hydrogen bonding between side chains occurred in a few cases that involved N25, R61, and K65. To examine whether interactions of these side chains are physiologically relevant, we introduced the exchanges N25A, R61A, and K65A. As R61 can also form the only salt bridge in this interface, we also analyzed an R61Q exchange, which removes the charge but still offers polar side chain properties. Similarly, we also included a K65L exchange in our analyses, which removes the charge while still offering aliphatic surfaces. As none of the single exchanges had any effect on LIN functionality, we finally generated an N25A/R61A double exchange variant, which also had no effect on function, indicating that interface 2 is not physiologically relevant (Table 2).

These functional data prompted us to analyze the conservation of residues in RI orthologs from T4 and related phages (Figure 3D). We found that the region covering interface 1 residues is more conserved than other regions of RI (marked with a yellow bar in Figure 3D). All seven interface 1-exposed residues whose mutation affected LIN are highly conserved, with almost always an F or Y at position 30, a hydrophobic residue (M, V, A, I, and F) at position 34, a Y strictly conserved at position 42, an S strictly conserved at position 53, an E almost always at position 54, an F or Y always at position 56, and an aromatic residue (Y, F, and W) almost always at position 57. The only interface 1 residue whose alanine mutation did not have an effect, D31, is at the border of interface 1 and not conserved (can be D, N, T, K, E, and H), which explains why the mutation did not have an effect. In contrast, none of the three side chains in interface 2 is conserved, further supporting the view that only interface 1 is relevant for physiological function (Figure 3D).

Based on sequence conservation aspects, we also examined the exchanges V41A, V41G, G79A, Y86A, and Y86L (highlighted in green in Figure 3D). V41 is a highly conserved residue close to interface 1 that neighbors the strictly conserved and essential Y42 of interface 1, but V41 is directed inward and thus not contacting the holin. G79 is a strictly conserved residue in the region of the conserved disulfide residues and therefore could potentially have been essential for LIN, and Y86 is at a position with a strictly conserved aromate (Y or F) near the C-terminus of RI. Importantly, none of the five mutations (V41A, V41G, G79A, Y86A, and Y86L) affected LIN, in agreement with the view that only the identified interface 1 residues inhibit holin (Table 2).

**Figure 4:**
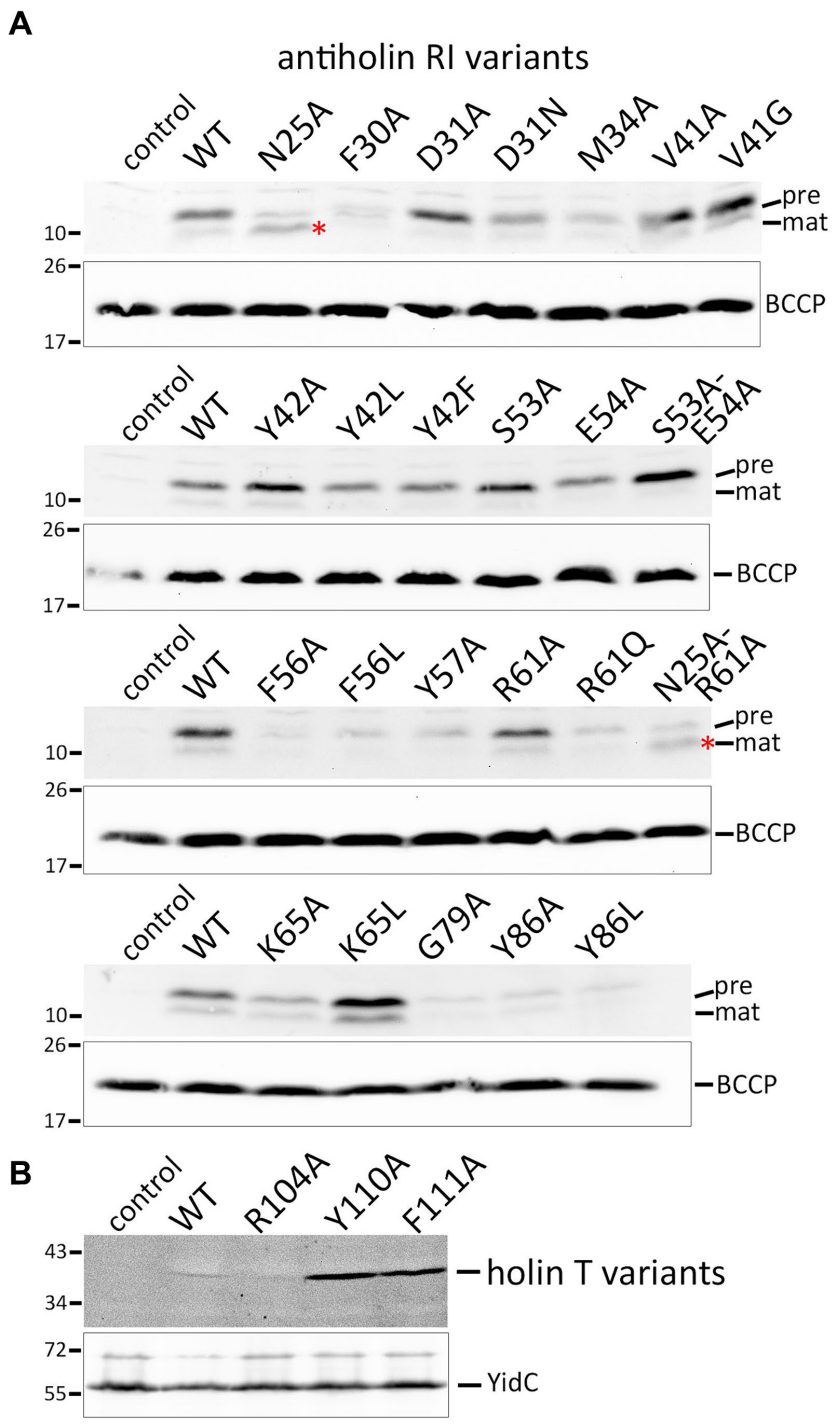
Detection of mutated protein variants. **(A)** SDS-PAGE/Western blot detection of all studied mutated RI variants in whole cell extracts. Positions of precursor (pre) and mature (mat) forms are indicated. Note that the N25A exchange immediately behind the signal peptidase cleavage site significantly improved the cleavage (red asterisks). The empty vector and wild-type RI were used as controls. Below the RI blots, blots with detections of BCCP are shown for the same fractions as loading controls. **(B)** Western blot with membrane fractions after production of holin T wild-type and indicated variants. Detections of the unrelated membrane protein YidC are shown as loading controls in the lower blot. Note that the holin-inactivating Y110A and F111A exchanges resulted in significantly more abundant protein, most likely because these variants did not affect the energization of the cells.

As it was in principle possible that loss-of-function effects were caused by the instability of the mutated protein variants, we detected the respective proteins using Western blotting (Figures 4A,B). The Y42A and Y42L exchanges, as well as the S53A/E54A double exchange at interface 1, all of which abolished LIN, had no negative effect on the stability of RI in direct comparison to wild-type RI. The F30A, M34A, F56A, and Y57A mutations, which also affected LIN, caused less abundant RI. However, this reduction cannot have caused the LIN defect, as the abundance of G79A, Y86A, and Y86L variants was similarly reduced without any effect on LIN. In this sense, the low abundance of G79A, Y86A, and Y86L variants served as important controls that demonstrated that the change in LIN functionality was not due to a low protein concentration. The holin variants Y110A and F111A, which strongly affected the lysis function, were significantly more abundant than the wild type and the R104A variant (Figure 4B). Most likely, these inactive holin variants did not affect membrane integrity and energization and therefore could be produced at higher levels, whereas functional holins could only be produced with lower yields.

The signal peptide is cleaved between A24 and N25. Interestingly, the Western blot analyses of the strains producing the RI(N25A) or the RI(N25A-R61A) variants showed that RI was more efficiently processed when N25 was mutated to an alanine. This clear enhancement of processing by the N25A exchange fully agrees with our previous hypothesis that nature designed the cleavage site of RI to be inefficient because membrane-anchored RI is needed for LIN.

In summary, our data indicate that only the conserved interface 1 mediates LIN, and therefore the physiologically relevant complex is a holin–antiholin dimer.

### 3.3 Signal peptide cleavage and degradation pathways determine the abundance of precursor and mature forms of RI

**Figure 5:**
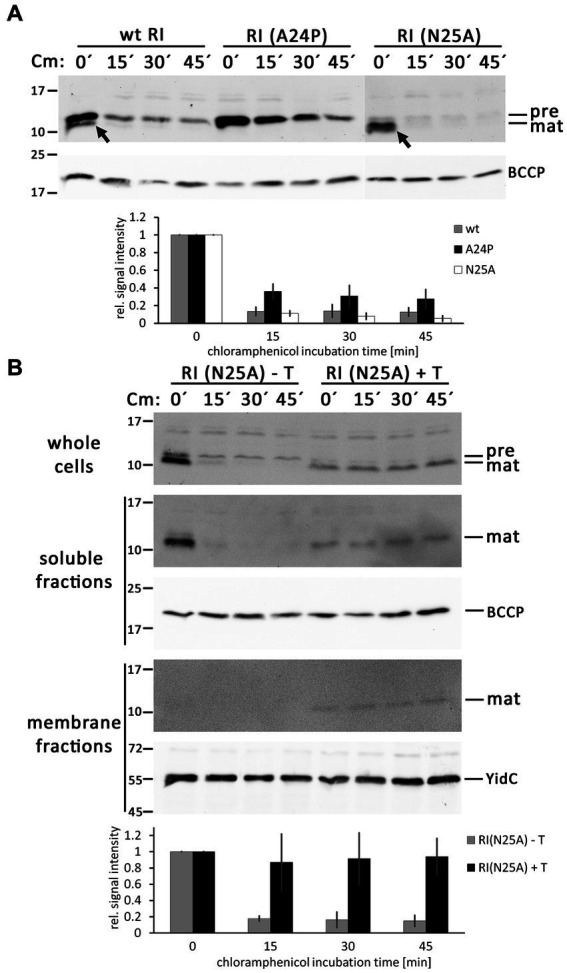
RI is rapidly degraded after signal peptide cleavage in the absence of the holin T. **(A)** Abundance of precursor and mature forms of wild-type (wt) RI and the mutated variants RI(A24P) and RI(N25A) in the absence of the holin T, analyzed by SDS-PAGE/Western blotting at indicated time points (0, 15, 30, and 45 min) after the addition of the translation inhibitor chloramphenicol. The diagram below shows the total RI signal intensity quantifications as obtained with three independent replicates, corrected according to loading controls, and normalized to the respective signal intensities at time point “0 min.” Error bars indicate standard deviations. **(B)** Same analysis as in **(A)**, but carried out with RI(N25A) in the absence (empty vector) or presence of the holin T. Shown are the blots for the whole cell fractions (upper blot), the soluble fractions (middle blots), and the membrane fractions (lower blots). Note that RI is stabilized in the presence of holin. See the text for more details. Less RI is formed, possibly due to the effects of the holin on the overall energization of the cells. Detections of BCCP or YidC are shown as loading controls. The diagram below displays the quantification of the whole cell fraction signals for this experiment, derived from three independent replicates as described in **(A)**.

To analyze the question of what determines the abundance of precursor and mature forms of RI in more detail, we inhibited translation by the addition of chloramphenicol and followed the fate of the corresponding bands over time using Western blotting (Figure 5A). With wild-type RI, the weak band of the mature protein rapidly disappeared, confirming that processed RI is subject to rapid proteolytic degradation. The stronger band of mature RI found with the optimized signal peptide cleavage site variant RI(N25A) was also rapidly degraded, showing that the higher abundance was only due to more efficient signal peptide cleavage and not to higher protease resistance. As a control, we also analyzed the RI(A24P) variant, which was not processed and more slowly degraded in the membrane, indicating that the decrease of precursor abundance in the wild type is due to signal peptide cleavage. A weak band of precursor that remained in the membrane at later times was possibly due to incomplete translational inhibition. The observed rapid degradation of RI in the periplasm aligns with findings from the Ry Young group, which identified DegP as the responsible periplasmic protease (Tran et al., 2007). As binding of a partially hydrophobic and positively charged C-terminus of proteins to the PDZ1-domain of DegP is crucial for the stimulation of proteolysis (Kim et al., 2011), we would like to note that the degradation kinetics likely also explains why the tested hexahistidine-tagged RI is less stable than the HA-tagged RI that was analyzed in a previous study (Mehner-Breitfeld et al., 2021). In any case, the observed mature form is very transient, and the low-abundant mature RI therefore reflects rapid turnover.

As binding of RI to the holin could protect RI from degradation, which would influence the abundance of precursor and mature forms of RI, we repeated these analyses with the RI(N25A) variant, which is rapidly processed in the absence of the holin, and specifically analyzed potential effects of the co-produced holin (Figure 5B). Notably, the presence of the holin stopped degradation of the mature RI. A portion of the mature RI fractionated with the membranes in a holin-dependent manner, indicating that the holin had bound to and stabilized RI. Mature RI, which had not degraded, was also detected in the soluble fraction. Mature RI was also detected in the soluble fraction, which is most likely due to some release of the not membrane-integral RI from the membrane during cell disruption. These interesting side aspects open new perspectives for future studies.

### 3.4 The identified relevant interaction and the dimeric structure of T/RI are also supported by AlphaFold2

**Figure 6:**
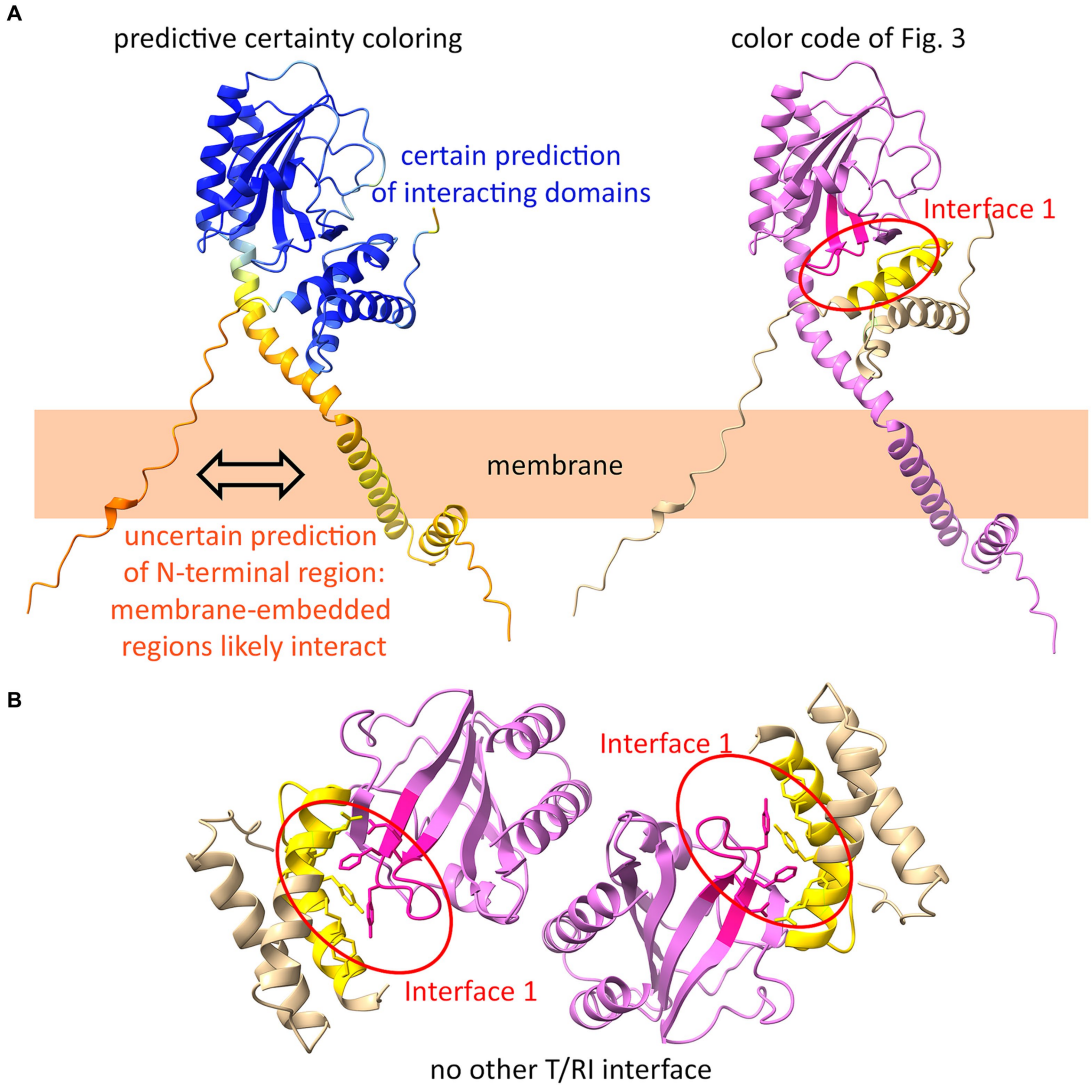
AlphaFold2 predicts interface 1 as the only interface. **(A)** AlphaFold2-model of a T/RI dimer predicts interface 1 of the T/RI crystal structure (as shown in Figure 3). In the structure on the left, the certainty of the prediction is colored from blue (certain) to orange (uncertain). On the right, the same structure is shown with the color code of Figure 3. Note that the membrane anchors most likely interact, which cannot yet be predicted by AlphaFold2. **(B)** When forced to predict a tetramer, AlphaFold2 predicts two T/RI dimers, each with only an interface 1.

AlphaFold2 is a highly accurate program for predicting protein structures. For membrane proteins, such as polytopic membrane proteins like ABC transporters, structure predictions are quite reliable (Tordai et al., 2022). However, there is no known holin or antiholin structure that includes the transmembrane domains, and certainly also no structure of the complex between membrane-anchored antiholins and full-length holins. As AlphaFold2 permits the prediction of oligomers, we used it to generate a holin–antiholin dimer that included all domains of the proteins. AlphaFold2 can accurately predict the soluble periplasmic domains of RI and holin T, which has been shown by overlays with subunits of the tetrameric crystal structure (Abeysekera et al., 2022). The predicted dimer interacted at interface 1, supporting our conclusion that only this interface is relevant (Figure 6A). When we forced AlphaFold2 to generate a tetramer, the resulting structure did not show any other holin–antiholin interface beyond interface 1, which fully agrees with our data (Figure 6B).

## 4 Discussion

The mechanism by which phages delay lysis in environments of high phage titer has been studied since the discovery of LIN by the *Escherichia coli* phage T4 in the 1940s (Hershey, 1946), and breakthroughs were the identification of the antiholins RI and RIII, and the structural characterization of the antiholin–holin interaction using X-ray crystallography (Ramanculov and Young, 2001a; Chen and Young, 2016; Krieger et al., 2020). Each of the protomers is synthesized with an N-terminal transmembrane helix. The tetrameric structure was solved only for the folded periplasmic domains, excluding the transmembrane helices, and in the case of the holin, a larger region that connects the transmembrane helix with the folded domain was also missing in the structure. It was believed that all four protomers were membrane-anchored. In the original crystal structure-based model, the positions of the four transmembrane helices were hypothetical, and only the postulation of four spatially separated transmembrane helices was a feasible option—something very unusual for a membrane protein complex (Krieger et al., 2020). Furthermore, an extreme kink of the holin linker region would be required to achieve access of the holin transmembrane helix to the membrane, and the structures did not provide supporting evidence for such a kink. Based on our finding that the transmembrane anchor of RI represents a cleavable signal peptide, a simplified tetramer model with only two transmembrane helices was proposed (Mehner-Breitfeld et al., 2021). Later AlphaFold2 predictions also supported the new model, as the assumed kink of the tetramer model was not predicted (Abeysekera et al., 2022). However, our functional analyses now show that—unexpectedly—the antiholin functions with its membrane anchor, as the abolishment of signal peptide cleavage did not interfere with LIN (Figure 1). We, therefore, had to question the tetramer model, and more detailed mutational analyses of the two holin/antiholin interaction sites in the tetramer indicated that only the larger interaction site 1 is required for LIN (Table 2), which is also supported by analyses of the conservation on sequence level (Figure 3D), and by AlphaFold2-modeling of dimeric or tetrameric associations (Figure 6). In conclusion, our data suggest that the physiologically relevant structure is a dimeric holin/antiholin association, and this dimeric structure now explains how the antiholin and the holin can interact in a membrane-anchored state to achieve LIN. Our dimeric model also does not demand any kink in the N-terminal region next to the structurally solved periplasmic domain of the holin, and thus the model agrees with the previous AlphaFold2-analysis (Abeysekera et al., 2022), and our own AlphaFold2-prediction of the heterodimer confirms this (Figure 6). The heterogeneity of interactions at the physiologically irrelevant interface 2, which are mainly interactions of backbone positions and non-conserved side chains, suggests that these associations have been promoted by crystallization conditions. The crystal structure of the holin–antiholin tetramer thus gave molecular insight into the natural holin–antiholin dimer, and the tetramer rather represents a crystallization-induced dimer of dimers (Krieger et al., 2020). We would like to emphasize that a previous biochemical analysis of the holin–antiholin association already indicated a heterodimer formation with the membrane-anchored components, as evidenced by formaldehyde cross-linking (Ramanculov and Young, 2001a), and with the soluble domains in solution, as evidenced by analytical ultracentrifugation (Moussa et al., 2012). However, the analytical ultracentrifugation in the later crystallographic study was believed to have been misleading and explainable by a possible unusual sedimentation behavior or the assumed tetramer (Krieger et al., 2020).

Interestingly, we also found that holin residues at interaction site 1 are not only required for antiholin interactions but also for the holin function. As membrane permeabilization by holins is mediated by holin multimerization (Young, 2014), our data suggest that the antiholins inhibit holin function by directly binding to the holin–holin interaction site. Therefore, antiholin RI functions by sterically blocking the holin–holin interactions. How holins interact with each other and how exactly they permeabilize the membrane has not been understood so far, but our identification of a likely holin–holin interaction site will hopefully result in further efforts and ideas to solve these issues. In many other phages, antiholins function as membrane integral proteins. Studied examples are the antiholin LysA for phage P2, which is a membrane protein with four transmembrane helices (To et al., 2013), and the antiholin S107 of phage lambda that contains two transmembrane helices (White et al., 2010). S107 is encoded by the same gene as the holin but uses another start codon that adds two residues to the N-terminus (Met-Lys). This extension prevents the insertion of the first transmembrane helix of the holin, and the two transmembrane helices of the antiholin suppress holin function (White et al., 2010).

Another highly interesting aspect is the fact that in the case of RI, a low-efficiency Sec signal peptide cleavage is used to regulate membrane protein function. To our knowledge, there is no other known example of such a regulation. The sequence analysis showed that the cleavage site is not conserved and atypical, lacking the usually seen A-X-A motif. Instead, for example, larger residues are found at positions −1, −3, or +1 that may reduce efficiency, and proline residues that surround the site most likely inhibit alternative cleavage sites. RI from phage T4 has an I-E-A↓N cleavage site, and our analyses already showed that an I-E-A↓A site facilitates processing (Figures 4, 5). It may well be that slow RI signal peptide cleavage kinetics are advantageous, as this can increase the probability of contacting the holin at the membrane surface. In the case of SAR domains of pinholins, the release of a non-cleaved Sec signal membrane anchor activates endolysin functions (Xu et al., 2005; Park et al., 2007), whereas the cleavage of the Sec signal membrane anchor of RI most likely reduces the function. Notably, in the case of RI, the slow processing does not abolish the holin interaction, nor does the holin interaction block the processing of the RI (Figure 5). Therefore, the induction of holin function appears to be unrelated to the cleavage of the RI signal anchor.

**Figure 7:**
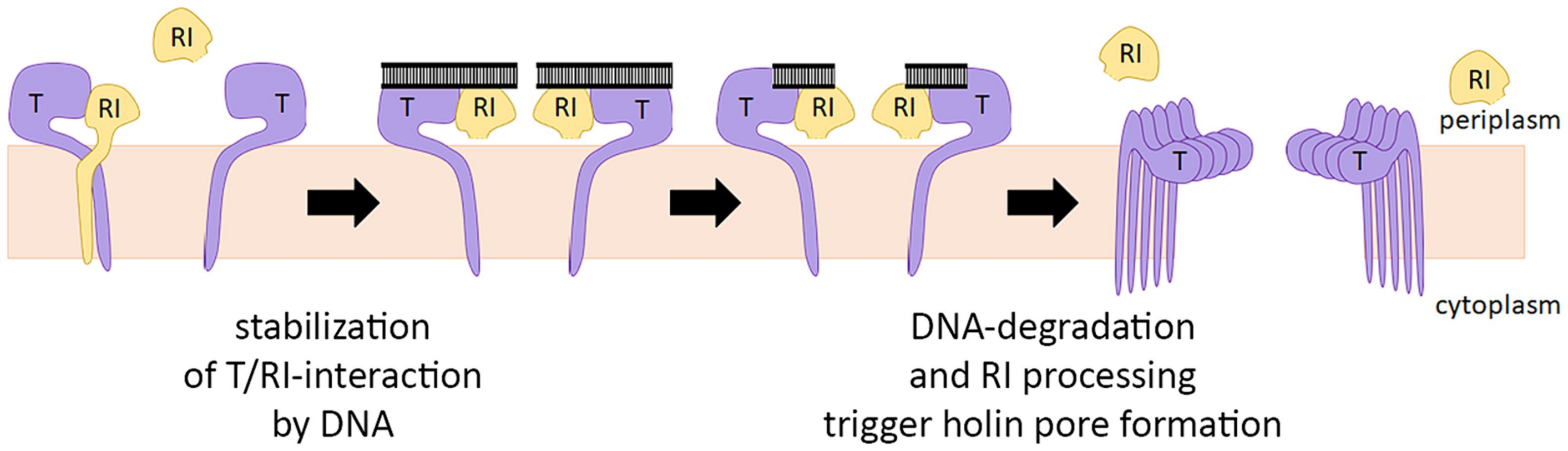
Revised model for the regulation of T4 lysis. The antiholin RI binds the holin T before the signal peptide is cleaved by forming a T/RI dimer. RI signal peptide is slowly cleaved, but this cleavage does not disrupt the interaction with the holin. Lysis timing depends on the concentration of active RI in the membrane. If not bound to holin T, signal peptide cleavage releases RI slowly into the periplasm, where RI is degraded. Periplasmic DNA likely stabilizes the T/RI dimer, thereby delaying lysis.

In summary, it can be concluded that the antiholin can inhibit the holin before the signal peptide is cleaved, and a heterodimer is formed in which both partners are membrane-anchored. Signal peptide cleavage of the antiholin does not alleviate the holin interaction (Figure 5B), and therefore the slow cleavage likely only facilitates the initial interaction at the membrane. The current model for the regulation of T4 lysis, therefore, must be modified (Figure 7). It is a single antiholin whose periplasmic domain forms a complex with individual holin subunits, inhibiting multimerization of holins by interacting with the same binding site responsible for holin–holin interactions. We could observe LIN without any periplasmic DNA, but we want to emphasize that high concentrations of RI were necessary to reconstitute LIN in these assays. Superinfections and the resulting periplasmic DNA can be expected to enhance the RI function to permit LIN at physiological concentrations, as it was previously shown that the complex binds DNA (Krieger et al., 2020).

## Data Availability

The original contributions presented in the study are included in the article/Supplementary material, further inquiries can be directed to the corresponding author.
