# Supplementary material for: A dimeric holin/antiholin complex controls lysis by phage T4
Source: Front Microbiol. 2024 Sep 5;15:1419106. doi: 10.3389/fmicb.2024.1419106 (PMC11413866; doi:10.3389/fmicb.2024.1419106)

frontiers

Supplementary Material

A dimeric holin/antiholin complex controls lysis by phage T4

Jan Michel Frederik Schwarzkopf, Denise Mehner-Breitfeld, Thomas Brüser*

*** Correspondence:** Thomas Brüser: brueser@ifmb.uni-hannover.de

Supplementary Table S1. Clustal Omega (1.2.4) multiple sequence alignment of the 168 RI antiholin homologues that has been used for generation of the WebLogo Figure 3D. RI of phage T4 is highlighted in yellow. (assignment by GenBank accession number; see Table S2 for phage names and hosts)

```
QPI17802.1          -----------------MIKH---ILA---ITL-L-----FSVSTVNAEVPKNFDSYVTA
QFR55726.1          ----------------MNIKR---LLQTSTLLF-LLSPVHGFTSESNRLVPVEFDQYINA
YP_009607330.1      ----------------MNIKR---ISMAVLFGLLMMTPT--THAADR-PVQKEFDDYINA
URQ04387.1          ---------MSKRVIVMNIKR---ISMAVLFGLLMMTPT--THAADR-PVQKEFDDYINA
YP_009194379.1      ----------------MNIKR---ISMAVLFGLLMMTPT--THAADR-PVQKEFDDYINA
YP_009607052.1      ---------MSKRVIVMNIKR---ISMAVLFGLLMMTPT--THAADR-PVQKEFDDYINA
YP_003579989.1      ---------MSKRVIVMNIKR---ISMAVLFGLLMMTPT--THAADR-PVQKEFDDYINA
YP_007348772.1      ----------------MNIKR---ISMAVLFGLLMMTPT--THAADR-PVQKEFDDYINA
UIW11501.1          ----------------MNIKR---ISKALMFGLLILSPS--SHADDRSVVAKEFDYYINA
DAG47327.1          ----------------MNIKR---ISKALMFGLLILSPS--SHADDRSVVAKEFDYYINA
YP_009194955.1      ----------------MNIKR---ISQALMFGLLMLSPA--SHADDR-VVPKEFDYYINA
YP_003858432.1      ----------------MNIKR---ISKALMFGLLILSPA--SHADD-SVVPKEFDYYINA
YP_009097745.1      ----------------MNIKR---ISKALMFGLLILSPA--SQADDRSVVAKEFDYYINA
YP_006986407.1      ----------------MNIKR---ISKALMFGLLILSPS--SHADDRSVVAKEFDYYINA
QUL77064.1          ----------------MNIKR---ISKALMFGLLILSPS--SHADDRSVVAKEFDYYINA
UIW12440.1          ----------------MNIKR---ISQAMMFGLLILSPA--SHADRNTVVPKEFDYYINA
QJI53183.1          ----------------MNIKR---ISQAMMFGLLILSPA--SHADRNTVVPKEFDYYINA
YP_009205796.1      ----------------MGIFR--TVACI--LMIGCLSPS---VSLANKSDPKLMDAFASS
UPW37817.1          ----------------MGIFR--TVACI--LMIGCLSPT---VSLANKSDPKLMDAFASS
CAI9865938.1        ----------------MGIFR--TVACI--LMIGCLSPT---SSLANKSDPKLMDAFASS
YP_001595227.1      ----------------MGIFR--TVACI--LMIGCLSPT---SSLANKSDPKLMDAFASS
WPJ21437.1          ----------------MDIFR--TAACI--LMAGCLSPT---VSLANNTNDKLMDAFASS
QAY00131.1          ----------------MDIFR--TAACI--LMAGCLGPS---VSLANDTNDKLMDAFASS
YP_003734244.1      --------------MCMDIFR--TAACI--LMAGCLGPS---VSLANDTNDKLMDAFASS
QBP35713.1          ----------------MDIFR--TAACI--LMAGCLSPT---VSLANDTNDKLMDAFASS
YP_009126310.1      ----------------MALRA---LFLSALIGIMCVP-----VHA--DTFNPKFDEYFEG
YP_007501144.1      -MTAQTKHWTKIRRPIMALRA---LFLSALIGIMCVP-----VHA--DTFNPKFDEYFEG
YP_009286464.1      ----------------MALRA---LFLSALIWILSIP-----AQA--DVFNPKFDEYFEG
YP_009615586.1      ----------------MALRA---LFLSALIGIMCVP-----VHA--DTFNPKFDEYFEG
QQG32244.1          ----------------MALKS---LFLSALMGIMLIP-----SSYADVQINSKFDEYFEG
WKV23456.1          ----------------MALKA---LALSALIGIMTIP-----ASYAEVDFNPKFDEYFEG
YP_009146546.1      ----------------MALKA---LALSALIGIMMIP-----ASYAEVNFNPKFDEYFEG
YP_009203826.1      ----------------MALKA---LALSALIGIMMIP-----ASYAEVDFNPKFDEYFEG
YP_004010271.1      ----------------MKSIL-----FFVMLSISCGI--------AANPVTKEFNSYVEG
YP_006488804.1      ----------------MKSIL-----LLCAFI-LCLM-----SS-AAQANDKEFNNFVEG
YP_009882281.1      ----------------MKAIL-----ISAMLL-GISF-----QQCQAA-DDKDFNRFVEG
UQS93713.1          ----------------MKAVL-----LFAMLV-GCSI-----QQCLAAEEDKDFDKFVEG
YP_009880905.1      ----------------MKTVL-----LFAMLV-GCSI-----QQCLAAEEDKDFDKFVEG
YP_009881568.1      ----------------MKTVL-----LFAMLV-GCSI-----QQCLAAEGDKDFDKFVEG
YP_009889763.1      ----------------MKAVL-----LFAMLV-GCSI-----QQCLAAEEDKDFDKFVEG
QKN88074.1          ----------------MKAVL-----LFAMLV-GCSI-----QQCLAAEGDKDFDKFVEG
YP_009886406.1      ----------------MKAVL-----LFAMLV-GCSI-----QQCLAAEEDKDFDKFVEG
YP_004300701.1      ----------------MKWVI-----MMLT--ITVSL-----A--CSAKNPDKFNTYFEG
YP_004009500.1      ----------------MKYLI-----FMIL--IVPSL-----CF-ASTEKPVKFDKYFEG
YP_010093716.1      ---------MAEFKNYFSPGI---I---ACVLTLCIG-----FSSAYASDDKYFNVYAEG
QPB12267.1          ----------------MSPIK---I---LVFLCLFAL-----PALGSTSDEKQFQEYAEG
YP_009211536.1      ----------------MDFTK---ICKAIVFFTACSM------SSISSADDTDYKSYVEG
QMP18845.1          MLRLQSVIMTNNKESFMDLIR---GLSLSLLLMGCLG------PNMSSATDGKFTQYADS
YP_004063793.1      ----------------MDLIR---GLSLSLLLMGCLG------PNLSSATDGKFTQYADS
QQG30800.1          MLRLQSVIMTNNKESFMDLIR---GLSLSLLLMGCLG------PNLSSATDGKFTQYADS
QUL77647.1          ----------------MDLIR---GLSLSLLLMGCLG------PNMSSATDGKFTQYADS
YP_009209851.1      ----------------MDLIR---GLSLSLLLMGCLG------PNMSSATDGKFTQYADS
UHS65324.1          ----------------MDLIR---GLSLSLILLGCLG------PNLSSATEGKFTQYADS
QIN95484.1          ----------------MDLIR---GLSLSLIILGCLG------PNLSSAAEGKFTQYADS
YP_003934732.1      ----------------MDLIR---GLSLSLIILGCLG------PNLSSATDGKFTQYADS
QWQ55747.1          ----------------MDLIR---GLSLSLIILGCLG------PNLSSATEGKFTQYADS
WCZ66167.1          -----------------MSFR---LATICILAGALFA------PHFSVASDESFDQYASG
YP_010090811.1      ----------------MGLIR---VLSTTILIGACLA------PSVSSAQDASFNQYVEG
YP_010092259.1      ----------------MGLIR---VLSTTILIGACLT------PSVSSAEDASFNQYVEG
UYM28759.1          ----------------MGLIR---VLSTAILIGACLA------PSVSSAEDASFNQYVEG
```

```
YP_009609435.1      ----------------MGLIR---VLSTTILIGACLA------PSVSSAEDASFNQYVEG
YP_009883816.1      ----------------MSLIRKTALACSVGLFLTFSM-------PISNAAEGNFKDYADG
YP_009030155.1      ----------------MSLIRKTALACSVGMFLTFSM-------PISNAAEGNFKDYADG
UES35696.1          -----------------MTLR--ALAAILFAAT--LI-------SPVSAEEANFEQYADG
YP_009005374.1      -----------------MTLR--ALAAILFAAT--LI-------SPVSAEEANFEQYADG
YP_004009968.1      -----------------MTLR--ALAAILFAAT--LI-------SPVSAEEANFEQYADG
WJZ27985.1          ---------------MALLLR--TFAALLISVTALSA-------VPSSANTGDFNEYLEG
QPI14021.1          ----------------MVLIR--TLVASLLAFSVFSV-------VPSSANTGDFTEFADG
YP_010090638.1      ----------------MLRYL---LICVLLVFASFSS-----SPR----GAEAKDSYFEG
YP_009850289.1      ----------------MAFKR-----SLCLALGVCIS-----LPVIAT-ETEGFNQYVEG
YP_009836720.1      ----------------MAFKH-----SLCLAVGVCIS-----LPVVAT-ETEGFNQYVEG
WKW88073.1          ----------------MAFKH-----SLCLAVGVCIS-----LPVVAT-ETEGFNQYVEG
QPB08927.1          ----------------MAFKP---I-CLALVLGCGIS-----LPAASH-DISNFNSYVEG
QEG11753.1          ----------------MAFKP---I-CLALVLGCGIS-----LPAASHNDISDYNSYVEG
YP_009190675.1      ----------------MAFKP---I-CLALVLGCGIS-----FPAASHDDISDYNSYVEG
WPH67859.1          ----------------MAFNH---I-CLALVLGCGIS-----FPAASHDDISDYNSYVEG
YP_009289456.1      ----------------MAFNH---I-CLALVLGCGIS-----FPAASHDDISDYNSYVEG
UGO47466.1          ----------------MAFNH---I-CLALVLGCGIS-----FPAASHDGISDYNSYVEG
QPX73789.1          ----------------MAFNH---I-CLALVLGCGIS-----FPAASHDDISDYNSYVEG
UNY41189.1          ----------------MAFNH---I-CLALVLGCGIS-----FPAASHNDISDYNSYVEG
UGO48449.1          ----------------MAFNH---I-CLALVLGCGIS-----LPAASHDDISDYNSYVEG
UMM76594.1          ----------------MAFNH---I-CLALVLGCGIS-----LPAASHNDISDYNSYVEG
UGO49742.1          ----------------MAFNH---I-CLALVLGCGIS-----LPAASHDDISDYNSYVEG
YP_010089418.1      ----------------MAFNH---I-CLALVLGCGIS-----FPAASHDDISDYNSYVEG
YP_009288785.1      ----------------MAFNH---I-CLALVLGCGIS-----FPAASHDDISDYNSYVEG
UOK17715.1          ----------------MAFNH---I-CLALVLGCGIS-----FPAASHDDISDYNSYVEG
UGO53259.1          ----------------MAFNH---I-CLALVLGCGIS-----FPAASHNDISDYNSYVEG
WAB00422.1          ----------------MAFNH---I-CLALVLGCGIS-----FPAASHNDISDYNSYVEG
QYC52795.1          ----------------MAFNP---I-CLALVLGCGIS-----FPAASHNDISDYNSYVEG
QOI66588.1          ----------------MAFIR---LVCMTLFFSIGIS------PNV-HADEAAFKQYVEG
YP_010095043.1      ----------------MAFIR---LVCVALFFGVGFS------PSV-HADGEAFNQYVEG
YP_010089700.1      ----------------MSLTK---LAVCAMMIAFFVS------PTASMADEHNKHQYFEG
YP_007235940.1      --------------RTMSLTK---LAVCAIMITLFIS------PVVLSSDEHKRHQYFEG
YP_009200371.1      ----------------MSLTK---LAVCAIMITLFIS------PVVLSSDEHKRHQYFEG
AIT75019.1          ----------------MALKA---TALFAMLGLSFVL----SPSI-EANVDPHFDKFMES
YP_010071311.1      ---------------------------MPCLGLAFAL----SPPI-EANVDPHFDKFMES
QXV84950.1          ----------------MALKA---TALFAMLGLSFVL----SPSI-EANVDPHFDKFMES
WBY53229.1          ----------------MALKA---TALFAMLGLAFAL----SPPI-EANVDPHFDKFMES
QOQ37471.1          ----------------MALKA---AALFAMLGLAFVL----SPPI-EANVDPHFDKFMES
YP_010070761.1      ----------------MALKA---TALFAMLGLAFAL----SPPI-EANVDSHFDKFMES
WAE77443.1          ----------------MALKA---TALFAMLGLAFAL----STPI-EANVDPHFDKFMES
WBF79919.1          ----------------MALKA---TALFAMLGLSFAL----SLPI-EANVDPHFDKFMES
ULA51879.1          ----------------MALKA---TALFAMLGLAFAL----SPPI-EANVDPHFDKFMES
WAX13030.1          ----------------MALKA---AALFAMLGLAFVL----SPPI-EANVDPHFDKFMES
YP_010072394.1      ----------------MALKA---TELFAMLGLAFAL----SPPI-EANVDPHFDKFMES
YP_004415003.1      ----------------MALKA---TALFAMLGLAFAL----SPPI-EANVDPHFDKFMES
YP_010071150.1      ----------------MALKA---TALFAMLGLAFAL----SPPI-EANADPHFDKFMES
YP_010091143.1      ----------------MALKA---TALFAMLGLSFAL----SPPI-EANVDPHFDKFMES
YP_010073757.1      ----------------MALKA---TVLFAMLGLSFVL----SPSI-EANVDPHFDKFMES
YP_010070485.1      ----------------MALKA---TALFAMLGLSFAL----SPPI-EANVDPHFDKFMES
YP_010077370.1      ----------------MALKA---TALFAMLGLAFAL----SPLI-EANVDPHFDKFMES
UDY80501.1          ----------------MALKA---TALFAMLGLAFVL----SPPI-EANVDPHFDKFMES
QWV60450.1          ----------------MALKA---AALFAMLGLAFVL----SPPI-EANVDPHFDKFMES
YP_007004487.1      ----------------MALKA---TALFAMLGLAFAL----SPPI-EANVDPHFDKFMES
YP_009153709.1      ----------------MALKA---TALFAMLGLSFVL----SPPI-EANVDPHFDKFMES
UIU27783.1          ----------------MALKA---TALFAMLGLSFVL----SPSI-EANVDPHFDKFMES
URY11966.1          ----------------MALKA---TALFAMLGLSFAL----SPSI-EANVDPHFDKFMES
YP_002854065.1      ----------------MALKA---TALFAMLGLSFAL----SPPI-EANVDPHFDKFMES
YP_009614761.1      ----------------MALKA---TALFAMLGLSFVL----SPSI-EANVDPHFDKFMES
YP_010069670.1      ----------------MALKA---TALFAMLGLSFVL----SPSI-EANVDPHFDKFMES
YP_009110927.1      ----------------MALKA---TALFAMLGLAFVL----SPPI-EANVDPHFDKFMES
QXN75810.1          ----------------MALKA---TALFAMLGLTFVL----SPPI-EANVDPHFDKFMES
YP_006986660.1      ----------------MALKA---TALFAMLGLSFVL----SPSI-EANVDPHFDKFMES
YP_009277482.1      ----------------MALKA---TALFAMLGLAFAL----SPSI-EANVDPHFDKFMES
YP_009210294.1      ----------------MALKA---TALFAMLGLSFVL----SPPI-EANVDPHFDKFMES
YP_002854444.1      ----------------MALKA---TALFAMLGLSFVL----SPSI-EANVDPHFDKFMES
QXV79213.1          ----------------MALKA---TALFAMLGLSFVL----SPSI-EANVNPHFDKFMES
YP_010106139.1      ----------------MALKA---TALFAMLGLSFAL----SPSI-EANVDPHFDKFMES
QOC55302.1          ----------------MALKA---TTLFAMLGLSFVL----SPSI-EANVDPHFDKFMES
URY14984.1          ----------------MALKA---TALFVMLGLSFVL----SPSI-EANVDPHFDKFMES
WJJ57515.1          ----------------MALKA---TALFAMLGLSFVL----SPSI-EANVDPHFDKFMES
NP_049717.1         ----------------MALKA---TALFAMLGLSFVL----SPSI-EANVDPHFDKFMES
QBO63621.1          ----------------MALKA---TALFAMLGLAFVL----SPSI-EANVDPHFDKFMES
UPW39297.1          ----------------MALRA---MALLAMLGLFVAT----TPFVSTAYVDPHFDNYMES
QEG06222.1          ----------------MALRA---IAVIAMLGFFAAT----TPIVGTAHIDPYFDNFMES
QBZ70948.1          ----------------MALRA---IAVMAMLGFFAAT----TPIVGTAYTDPYFDNFMES
YP_009056689.1      ----------------MALRA---IAIVAMLGFFAAT----TPIVGTAYVDPYFDNFMES
QSL99198.1          ----------------MALRA---IAIVAMLGFFAAT----TPIVGTAYVDPYFDNFMES
YP_009100645.1      -----------------------------MLGFFAAT----TPIVGTAYVDPYFDNFMES
QBP05720.1          ----------------MALRA---IAIVAMLGFFAAT----TPIVGTAYTDPYFDNFMES
AYR04018.1          ----------------MALRA---IAVMAMLGFFAAT----TPIVGTAYVDPYFDNFMES
YP_009111273.1      ----------------MALRA---IAVMAMLGFFAAT----TPIVGTAYVDPYFDNFMES
NP_861799.1         -----------------------------MLGFFAAT----TPIVGTAYVDPYFDNFMES
YP_010088590.1      ----------------MALRA---IAVMAMLGFFAAT----TPIVGTAYVDPYFDNFMES
WPK33788.1          ----------------MALRA---IAIVAMLGFFAAT----TPIVGTAYIDPYFDNFMES
QVW29258.1          ----------------MALRA---IAIVAMLGFFAAT----TPIVGTAYVDPYFDNFMES
QHR76563.1          ----------------MALRA---IAVMAMLGFFAAT----TPIVGTAYVDPYFDNFMES
WPK34047.1          ---------------------------MAMLGFFAAT----TPIVGTAYVDPYFDNFMES
QXV77046.1          ----------------MALRA---IVIVAMLGFFAAT----TPIVGTAYVDPYFDNFMES
```

```
UUB18216.1              ----------------MALRA---IAIVAMLGFFAAT----TPIVGTAYVDPYFDNFMES
YP_010096345.1          ----------------------------MLGFFAAT----TPIVGTAYVDPYFDNFMES
AWM11858.1              ----------------MALRA---IAVMVMLGFFAVT----TPIVGTAYVDPYFDNFMES
WBF80716.1              ----------------MALRA---IAVMVMLGFFAVT----TPIVGTAYIDPYFDNFMES
YP_009592859.1          ----------------MALRA---IAVMAMLGFFAAT----TPIVGTAYIDPYFDNFMES
YP_009037398.1          --------------------------MAMLGFFAAT----TPIVGTAYIDPYFDNFMES
YP_010100079.1          ----------------MALRA---IAVMAMLGFFAAT----TPIVGTARIDPYFDNFMES
WPK34318.1              ----------------MALRA---IAVIAMLGFFAAT----TPIVGIAHIDPYFDNFMES
YP_010088390.1          ----------------MALRA---IAVMAMLGFFAAT----TPIVGTAHIDPYFDNFMES
UOL50774.1              ----------------MALRA---IAVIAMLGFFAAT----TPIVGTAHIDPYFDNFMES
YP_010076184.1          ----------------MALRA---IAVIAMLGFFAAT----TPIVGTAHIDPYFDNFMES
QHR68209.1              ----------------MALRA---IAVIAMLGFFAAT----TPIVGTAHIDPYFDNFMES
QHR72707.1              ----------------MALRA---IAVIAMLGFFAAT----TPIVGTANIDPYFDNFMES
YP_009225157.1          ----------------MALRA---IAVIAMLGFFAAT----TPIVGTAHIDPYFDNFMES
CAH1486843.1            ----------------MALRA---IAVIAMLGFFAAT----TPIVGTAYIDPYFDNFMES
YP_010068772.1          ----------------MALRA---IAVMAMLGFFAAT----TPIVGTAYTDPYFDNFMES
YP_009608297.1          --------------------------MAMLGFFAAT----TPIVGTAYTDPYFDNFMES
YP_010246930.1          ----------------MALRA---IAVMAMLGFFAAT----TPNVGTAYTDPYFDNFMES
ULA52136.1              ----------------MALRA---IAVMAMLGFFAAT----TPNVGTAYTDPYFDNFMES
                                                                               :   .

QPI17802.1              A-LTVYLENIQPSVKTSELFYDYLKKNWTFD-KCFTN-MECQKLGVEVAKEFARNHT---
QFR55726.1              A-LRVYKATIPPSINTSEMFYDFMEKKWQQK-QCTDE-TDCRSLGMRVATEFANNYNVRD
YP_009607330.1          A-LQVYITTIPPSVNTSELFYNYMERKWRQK-QCRSE-SECAKLGIRVAYEFASIHRS--
URQ04387.1              A-LQVYITTIPPSVNTSELFYNYMERKWRQK-QCRSE-SECAKLGIRVAYEFASIHRS--
YP_009194379.1          A-LQVYITTIPPSVNTSELFYNYMERKWHQK-QCRSE-SECAKLGIRVAYEFASIHRS--
YP_009607052.1          A-LQVYITTIPPSVNTSELFYNYMERKWHQK-QCRSE-SECAKLGIRVAYEFASIHRS--
YP_003579989.1          A-LQVYITTIPPSVNTSELFYNYMERKWHQK-QCRTE-SECAKLGIRVAYEFARIHRS--
YP_007348772.1          A-LQVYITTIPPSVNTSELFYNYMERKWHQK-QCRTE-SECAKLGIRVAYEFASIHRS--
UIW11501.1              A-LQVYLDTIPPSVNTSELFYNYMERKWQQK-QCQTK-PECTKLGLRVANEFATIHRS--
DAG47327.1              A-LQVYLDTIPPSVNTSELFYNYMERKWQQK-QCQTK-PECTKLGLRVANEFASIHRS--
YP_009194955.1          A-LQVYLDTIPPSVNTSELFYNYMERKWQQK-QCKSE-PECKRLGLRVASEFAAIHRS--
YP_003858432.1          A-LQVYLDTIPPSVNTSELFYNYMERKWQQK-QCKSE-PECKRLGLRVATEFASIHRS--
YP_009097745.1          A-LQVYLDTIPPSVNTSELFYNYMERKWQQK-QCKSE-PECKRLGLRVATEFASIHRS--
YP_006986407.1          A-LQVYLDTIPPSVNTSELFYNYMERKWQQK-QCKSE-PECKRLGLRVASEFAAIHRS--
QUL77064.1              A-LQVYLDTIPPSVNISELFYNYMERKWQQK-QCKSE-PECKRLGLRVASEFAAIHRS--
UIW12440.1              A-LQVYLDTIPPSVNTSELFYNYMERKWQQK-QCRTE-PECKHLGYRVANEFATIHRS--
QJI53183.1              A-LQVYLDTIPPSVNTSELFYNYMERKWQQK-QCRTE-PECKHLGICYYSSFVRDERCKH
YP_009205796.1          A-MPVYAQMSKPSVKASEDYWAYVTASWKLS-NCNTI-ESCSIEGRAVGERYAKLMKVDD
UPW37817.1              A-MPVYAQMSKPSVKASEDYWAYVTASWKLS-NCNTI-ESCSIEGRAVGERYAKLMKVDD
CAI9865938.1            A-MPVYAQMSKPSVKASEDYWAYVTASWKIS-NCNTI-ESCSIEGQAIGERYAKLMKVDD
YP_001595227.1          A-MPVYAQMSKPSVKASEDYWAYVTASWKIS-NCNTI-ESCSIEGRAIGERYAKLMKVDD
WPJ21437.1              A-MPVYAQMSRPSVKASEDYWAYLNASWKNS-SCQTV-ETCSIEGRAIGERYAKLMKVED
QAY00131.1              A-MPVYAQMSRPSVKASEDYWAYVTASWKNS-SCRTI-EACSIEGRAIGERYAKLMKVED
YP_003734244.1          A-MPVYAQMSRPSVKASEDYWAYVTASWKNS-SCRTI-EACSIEGRAIGERYAKLMKVED
QBP35713.1              A-MPVYAQMSRPSVMASEDYWAYVTASWKNS-SCRTI-EACSIEGRAIGERYAKLMKVED
YP_009126310.1          A-LKVYTQYKIYNKRESEQFFSFVKSKWDRQ---PCT-STCEAAGVEVAKEYYTNRFTEK
YP_007501144.1          A-LKVYTQYKIYNKRESEQFFSFVKSKWDRQ---PCT-STCEAAGVEVAKEYYTNRFTEK
YP_009286464.1          A-LKVYTHYKIYNKQESEQFFSFVKSKWDSQ---SCA-ESCEAKGAEVAKQYYTNRLIEK
YP_009615586.1          A-LKVYTQYKIYNKRESEQFFSFVKSKWDSQ---SCN-ESCEAKGAEVAKQYYTNRLIEK
QQG32244.1              A-LKVYSQYKIYNKQESEQFFSFVKSKWESR---QCT-NNCEADGVRVGKEYYTNRMIDK
WKV23456.1              A-LKVYSQYKIYNKQESEQFFTFVKSKWERQ---PCT-NSCEADGALVAQEYYTNRLVEG
YP_009146546.1          A-LKVYSQYKIYNKQESEQFFTFVKSKWERQ---PCT-NNCEADGALVAQEYYTNRLVEG
YP_009203826.1          A-LKVYSQYKIYNKQESEQFFTFVKSKWERQ---PCT-NNCEADGALVAQEYYTNRLVEG
YP_004010271.1          A-LEVYSQFKRPSKAESERFYTFVQEKWNET---SCS-KSCSAEGYSVAKQYAKEKKVEI
YP_006488804.1          A-LEVYSQFKKPSKAESERFYSFLQAKWSSN---ECT-QNCSVAGYNTGKQYVKEKNVEI
YP_009882281.1          A-LTVYSQFKKPSKAESERFYDFVQAKWSKA---DCK-HDCSDVGYAIGQQYAKEKNIEI
UQS93713.1              A-LIVYSQFKTPSKQESERFYTFIQDRWNKS---ECS-RDCTHVGYVIGRQYVKEKNIEI
YP_009880905.1          A-LIVYSQFKTPSKQESERFYTFIQDRWNKS---ECS-RDCTHVGYVIGRQYAKEKNIEI
YP_009881568.1          A-LIVYSQFKTPSKQESERFYTFIQDRWNKS---ECS-RDCTHVGYVIGRQYAKEKNIEI
YP_009889763.1          A-LIVYSQFKTPSKQESERFYTFIQDRWNKT---ECS-RDCTHVGYVIGRQYAKEKNIEI
QKN88074.1              A-LIVYSQFKTPSKQESERFYTFIQDRWNKS---ECS-RDCTHVGYVIGRQYAKEKNIEI
YP_009886406.1          A-LIVYSQFKTPSKQESERFYTFIQDRWNKS---ECS-RDCTHVGYVIGRQYAKEKNIEI
YP_004300701.1          A-MTVYSQFENPSKDESERFYAFVKSKWTET---SCK-NDCTEWGQVTAKEYVKRKNIQV
YP_004009500.1          A-LEVYSQFETPSKKESEEFYVFVKAKWNKS---TCT-IDCDQWGYAAGQEYAKKKNIKI
YP_010093716.1          A-MSVYSKLKEPSKEESEQFYAFIRTKWEET---NCQNNKCSEDGKTAAQEYVYNLKKEL
QPB12267.1              A-LSVYAQIKEPSKEESERFYAYIKKRWEQS---NCTSTTCTEDGMTAAKEYVYVWNKEL
YP_009211536.1          A-LQVYKQFEEPSVKESQKFLNFVNTRWKDS-NEKCF-TACSLEGKNAAKDYASTNNIKL
QMP18845.1              A-MQIYSQFKEPSVAQSEQFWAFIKTEWNNKSQCVNE-ITCQIDGRAAAVEYAKLMKVKL
YP_004063793.1          A-MQIYSQFKEPSVEQSEQFWAFIKTEWNNKSQCVNE-ITCQIDGRAAAVEYAKLMKVKL
QQG30800.1              A-MQIYSQFKEPSVEQSEQFWAFIKTEWNNKSQCVNE-ITCQIDGRAAAVEYAKLMKVKL
QUL77647.1              A-MQIYSQFKEPSVEQSEQFWAFIKTEWNNKSQCVNE-ITCQIDGRAAAVEYAKLMKVKL
YP_009209851.1          A-MQIYSQFKEPSVEQSEQFWAFIKTEWNNKSQCVNE-ITCQIDGRAAAVEYAKLMKVKL
UHS65324.1              A-MKIYSQFKEPSVHQSEQFWAFIKTEWNNKSQCETE-ITCKSDGKAAAREYAKLMKVKL
QIN95484.1              A-MKIYSQFKEPSVHQSEQFWAFIKTEWNNKSQCETE-ITCKSDGKAAAREYAKLMKVKL
YP_003934732.1          A-MKIYSQFKEPSVHQSEQFWAFIKTEWNNKSQCETE-ITCKSDGKAAAREYAKLMKVKL
QWQ55747.1              A-MKIYSQFKEPSVHQSEQFWAFIKTEWNNKSQCETE-ITCKSDGKAAAREYAKLMKVKL
WCZ66167.1              A-LTVYKQFKEPSKEQSEQFLAFIKDKWVQE-TC----VSCHEDGINAGMEYAYRMKVPL
YP_010090811.1          A-LTVYSKFKEPSKQQSEKFFAFISAKWQEQ-EGECY-NNCSIDGKSAGQEYANRMKVPL
YP_010092259.1          A-LTVYSKFKEPSKQQSEKFFAFIQAKWQEK-DGVCD-RDCSVDGKHAGIEYAYSMKVPL
UYM28759.1              A-LTVYSKFKEPSKQQSEKFFAFIQAKWQEK-DGVCD-RDCSVDGKHAGIEYAYSMKVPL
YP_009609435.1          A-LTVYSKFKEPSKQQSEKFFAFIQAKWQEK-DGVCD-RDCSVDGKHAGIEYAYSMKVPL
YP_009883816.1          A-MLVYSKFKQPSKTESEQFYSYIKSRWERE---NCE--YCILPGKKAAEEYAVLRRVEL
YP_009030155.1          A-MLVYSKFKQPSKTESEQFYSYIKSRWERE---NCE--YCILPGKKAAEEYAVLRRVEL
UES35696.1              A-MVVYSKFKEPSKEESERFFSFIKQKWAAS---SCT-TQCTEEGVHAGKQYVSLTKVKL
YP_009005374.1          A-MAVYSKFKEPSKEESERFFSFIKQKWAAS---SCT-TQCTEEGVHAGKQYVSLTKVKL
YP_004009968.1          A-MAVYSKFKEPSKEESERFFSFIKQRWAAS---SCT-TQCTEEGVHAGKQYVSLTKVKL
WJZ27985.1              A-MSVYSKFKAPSVEESEQFYAFIKSKWQRD---DCS-TQCTADGKQAGAEYATLYQVNL
QPI14021.1              A-MSVYQKFTSPSVQESEQFFAFIKSKWQKD---DCS-NQCEADGKKAAKEYAALYRVNL
```

```
YP_010090638.1       A-LKIYSQFKEPSRLESEKFFEFVRIKWEQG---SCS-NNCTEQGKRAGMEYANLKKVEL
YP_009850289.1       A-LKVYAQFQKPSKQESEQFYSFIKNKWSTV---SCT-SECSVTGINAGLQYASQMRIPL
YP_009836720.1       A-LKVYAQFQKPSKQESEQFYSFIKNKWSTV---NCT-SECSVTGINAGLQYASQMRIPL
WKW88073.1           A-LKVYSQFQKPSKQESEQFYSFIKNKWSTV---NCT-SECSVTGINAGLQYASQMRIPL
QPB08927.1           A-LKVYAQFKEPSKQESEQFYGFLKSKWKSE---VCS-KNCDLVGRNAGVEYANRMRIQL
QEG11753.1           A-LQVYAKFKEPSKQESEQFYSFIQSKWKSE---SCS-KDCDSLGRSAGEEYANRMRIQL
YP_009190675.1       A-LQVYAKFKEPSKQESEQFYSFIQSKWKSE---SCS-KDCDSLGRSAGEEYANRMRIQL
WPH67859.1           A-LQVYAKFKEPSKQESEQFYAFXQSKWKSE---SCS-KDCDSLGRSAGEEYANRMRIQL
YP_009289456.1       A-LQVYAKFKEPSKQESEQFYAFVQSKWKSE---SCS-KDCDSLGRSAGEEYANRMRIQL
UGO47466.1           A-LQVYAKFKEPSKQESEQFYAFIQSKWKSE---SCS-KDCDSLGRSAGEEYANRMRIQL
QPX73789.1           A-LQVYAKFKEPSKQESEQFYAFIQSKWKSE---SCS-KDCDSLGRSAGEEYANRMRIQL
UNY41189.1           A-LQVYAKFKEPSKQESEQFYSFIQSKWNSE---SCS-KDCDSLGRSAGEEYANLMRIQL
UGO48449.1           A-LQVYAKFKEPSKQESEQFYSFIQSKWKSE---SCS-KDCDSLGRSAGEEYANRMRIQL
UMM76594.1           A-LQVYAKFKEPSKQESEQFYSFIQSKWKSE---SCS-KDCDSLGRSAGEEYANRMRIQL
UGO49742.1           A-LQVYAKFKEPSKQESEQFYSFIQSKWKSE---SCS-KDCDSLGRSAGEEYANRMRIQL
YP_010089418.1       A-LQVYAKFKEPSKQESEQFYSFIQSKWNSE---SCS-KDCDSLGRSAGEEYANRMRIQL
YP_009288785.1       A-LQVYAKFKEPSKQESEQFYSFIQSKWKSE---SCS-KDCDSLGRSAGEEYANRMRIQL
UOK17715.1           A-LQVYAKFKEPSKQESEQFYSFIQSKWKSE---SCS-KDCDSLGRSAGEEYANSMRIQL
UGO53259.1           A-LQVYAKFKEPSKQESEQFYSFIQSKWKSE---SCS-KDCDSLGRSAGEEYANRMRIQL
WAB00422.1           A-LQVYAKFKEPSKQESEQFYSFIQSKWKSE---SCS-KDCDSLGRSAGEEYANSMRIQL
QYC52795.1           A-LQVYAKFKEPSKQESEQFYSFIQSKWKSE---SCS-KDCDSLGRSAGEEYANSMRIQL
QOI66588.1           A-MAVYSKFKEPSKQESEQFYTFIKSKWQKE---TCI-SNCKNDGLKAGHEYAVRMNVEI
YP_010095043.1       A-MAVYSKFKEPSKQESEQFFTFIKSKWEKE---TCV-SQCKNNGQRAGIEYASVMKVEF
YP_010089700.1       A-MTIYSKFKEPSREQSEQFYSFIKSKWQQA---RCQ-ASCSVDGRDAANEYAMQRKVKL
YP_007235940.1       A-MSVYSKFKEPSKERSEQFYMFIKSKWVLA---KCN-STCSNDGVEAAKEYAVQRQVQL
YP_009200371.1       A-MSVYSKFKEPSKERSEQFYMFIKSKWVLA---KCN-STCSNDGVEAAKEYTVQRQVQL
AIT75019.1           GIRHVYMLFENKSVESSEQFYSFMRTTYKMT-RALLI-LNV-------------------
YP_010071311.1       GIRHVYMLFENKSVESSEQFYSFMRTTYKND-PCSSD-FECIERGAEMAHSYARIMNIKL
QXV84950.1           GIRHVYMLFENKTIESSEQFYSFMRTTYKND-PCSSD-FECIERGAEMAQSYARIMNIKL
WBY53229.1           GIRHVYMLFENKSVESSEQFYSFMRTTYKK------------------------------
QOQ37471.1           GIRHVYTLFENKSVESSKQFYSFMRTTYKND-PCSSD-FECIERGAEMAQSYARIMNIKL
YP_010070761.1       GIRHVYMLFENKSVESSEQFYSFMRTTYKND-PCSSD-FECIERGAEMAQSYARIMNIKL
WAE77443.1           GIRHVYMLFENKSVESSEQFYSFMRTTYKND-PCSSD-FECIERGAEMAQSYARIMNIKL
WBF79919.1           GIRHVYMLFENKSVESSEQFYSFMRTTYKND-PCSSD-FECIERGAEMAQSYARIMNIKL
ULA51879.1           GIRHVYMLFENKSVESSEQFYSFMRTTYKND-PCSSD-FECIERGAEMAQSYARIMNIKL
WAX13030.1           GIRHVYTLFENKSVESSEQFYSFMRTTYKND-PCSSD-FECIERGAEMAQSYARIMNIKL
YP_010072394.1       GIRHVYMLFENKSVESSEQFYSFMRTTYKND-PCSSD-FECIERGAEMAQSYARIMNIKL
YP_004415003.1       GIRHVYTLFENKSVESSEQFYSFMRTTYKND-PCSSD-FECIERGAEMAQSYARIMNIKL
YP_010071150.1       GIRHVYMLFENKSVESSEQFYSFMRTTYKND-PCSSD-FECIERGAEMAQSYARIMNIKL
YP_010091143.1       GIRHVYTLFENKSVESSEQFYSFMRTTYKND-PCSSD-FECIERGAEMAQSYARIMNIKL
YP_010073757.1       GIRHVYTLFENKSVESSEQFYSFMRTTYKND-PCSSD-FECIERGAEMAQSYARIMKIKL
YP_010070485.1       GIRHVYMLFENKSVESSEQFYSFMRTTYKND-PCSSD-FECIERGAEMAQSYTRIMNIKL
YP_010077370.1       GIRHVYMLFENKSVESSEQFYSFMRTTYKND-PCSSD-FECIERGAEMAQSYARIMNIKL
UDY80501.1           GIRHVYTLFENKSVESSEQFYSFMRTTYKND-PCSSD-FECIERGAEMAQSYARIMNIKL
QWV60450.1           GIRHVYMLFENKSVESSEQFYSFMRTTYKND-PCSSD-FECIERGAEMAQSYARIMNIKL
YP_007004487.1       GIRHVYMLFENKSVESSEQFYSFMRTTYKND-PCSSD-FECIERGAEMAQSYARIMNIKL
YP_009153709.1       GIRHVYTLFENKSVESSEQFYSFMRTTYKND-PCSSD-FECIERGAEMAQSYARIMNIKL
UIU27783.1           GIRHVYTLFENKSVESSEQFYSFMRTTYKND-PCSSD-FECIERGAEMAQSYARIMKIKL
URY11966.1           GIRHVYMLFENKSVESSGQFYSFMRTTYKND-PCSSD-FECIERGAEMAQSYARIMNIKL
YP_002854065.1       GIRHVYMLFENKSVESSEQFYSFMRTTYKND-PCSSD-FECIERGAEMAQSYARIMNIKL
YP_009614761.1       GIRHVYTLFENKSVESSEQFYSFMRTTYKND-PCSSD-FECIERGTEMAQSYARIMNIKL
YP_010069670.1       GIRHVYMLFENKSVESSEQFYSFMRTTYKND-LCSSD-FECIERGAEMAQSYARIMNIKL
YP_009110927.1       GIRHVYMLFENKSVESSEQFYSFMRTTYKND-PCSSD-FECIERGAEMAQSYARIMNIKL
QXN75810.1           GIRHVYMLFENKSVESSEQFYSFMRTTYKND-PCSSD-FECIERGAEMAQSYARIMNIKL
YP_006986660.1       GIRRVYMLFENKSVESSEQFYSFMRTTYKND-PCSSD-FECIERGAEMAQSYARIMNIKL
YP_009277482.1       GIRHVYMLFENKSVESSEQFYSFMRTTYKND-PCSSD-FECIERGAEMAQSYARIMNIKL
YP_009210294.1       GIRHVYMLFENKSVESSEQFYSFMRTTYKND-PCSSD-FECIERGAEMAQSYARIMNIKL
YP_002854444.1       GIRHVYTLFENKSVESSEQFYSFMRTTYKND-PCSSD-FECIERGAEMAQSYARIMNIKL
QXV79213.1           GIRHVYMLFENKSVESSEQFYSFMRTTYKND-PCSSD-FECIERGAEMAQSYARIMNIKL
YP_010106139.1       GIRHVYMLFENKSVESSEQFYSFMRTTYKND-PCSSD-FECIERGAEMAQSYARIMNIKL
QOC55302.1           GIRHVYMLFENKSVESSEQFYSFMRTTYKND-PCSSD-FECIERGAEMAQSYARIMNIKL
URY14984.1           GIRHVYMLFENKSVESSEQFYSFMRTTYKND-PCSSD-FECIERGAEMAQSYARIMNIKL
WJJ57515.1           GIRHVYMLFENKSVESSEQFYSFMRTTYKND-PCSSD-FECIERGAEMAQSYAKIMNIKL
NP_049717.1          GIRHVYMLFENKSVESSEQFYSFMRTTYKND-PCSSD-FECIERGAEMAQSYARIMNIKL
QBO63621.1           GIRHVYMLFENKSVESSEQFYSFMRTTYKND-PCSSD-FECIERGAEMAQSYARIMNIKL
UPW39297.1           GIKNVYSLFEIHNVENSEKFYSYMAKSFKAN-PCQDA-FECHEQGTRTARQFAEFMNIKL
QEG06222.1           GIKNVYTLFEIQNVENSEKFYKYMAKHYKNS-PCDDA-FECHEQGIKNCQTICRVHENKI
QBZ70948.1           GIKNVYTLFEIQNVENSEKFYKYMVKHYKNS-PCDDA-FECHEQGIKNCQTICRVHENKI
YP_009056689.1       GIKNVYTLFEIQNVENSEKFYNYMAKHYKNS-PCDDA-FECQEQGIKTARQFAEFMKIKL
QSL99198.1           GIKNVYTLFEIQNVENSEKFYKYMAKHYKNS-PCDDA-FECQEQGIKTARQFAEFMKIKL
YP_009100645.1       GIKNVYTLFEIQNVENSEKFYKYMAKHYKNS-PCDDA-FECQEQGIKTARQFAEFMKIKL
QBP05720.1           GIKNVYTLFEIQNVENSEKFYKYMVKHYKNS-PCDDA-FECHEQGIKTARQFAEFMKIKL
AYR04018.1           GIKNVYTLFEIQNVENSEKFYKYMAKHYKNS-PCDDA-FECHEQGIKTAKQFAEFMKIKL
YP_009111273.1       GIKNVYTLFEIQNVENSEKFYKYMAKHYKNS-PCDDA-FECHEQGIKTARKFAEFMKIKL
NP_861799.1          GIKNVYTLFEIQNVENSEKFYKYMAKHYKNS-PCDDA-FECHEQGIKTARKFAEFMKIKL
YP_010088590.1       GIKNVYTLFEIQNVENSEKFYKYMAKHYKNS-LCDDA-FECHEQGIKTARQFAEFMKIKL
WPK33788.1           GIKNVYTLFEIQNVENSEKFYKYMAKHYKNS-PCDDA-FECHEQGIKTARQFAEFMKIKL
QVW29258.1           GIKNVYTLFEIQNVENSEKFYKYMAKHYKNS-PCDDA-FECHEQGIKTARQFAEFMKIKL
QHR76563.1           GIKNVYTLFEIQNVENSEKFYKYMAKHYKNS-PCDDA-FECHEQGIKTARQFAEFMKIKL
WPK34047.1           GIKNVYTLFEIQNVENSEKFYKYMAKHYKNS-PCDDA-FECHEQGIKTARQFAEFMKIKL
QXV77046.1           GIKNVYTLFEIQNVENSEKFYKYMAKHYKNS-PCDDA-FECHEQGIKTARQFAEFMKIKL
UUB18216.1           GIKNVYTLFEIQNVENSEKFYKYMAKHYKNS-PCDDA-FECHEQGIKTARQFAEFMKIKL
YP_010096345.1       GIKNVYTLFEIQNVENSEKFYKYMAKHYKNS-PCDDA-FECHEQGIKTARQFAEFMKIKL
AWM11858.1           GIKNVYTLFEIQNVENSEKFYKYMAKHYKNS-PCDDA-FECHEQGIKTARQFAEFMKIKL
WBF80716.1           GIKNVYTLFEIQNVENSEKFYKYMAKHYKNS-PCDDA-FECHEQGIKTARQFAEFMKIKL
YP_009592859.1       GIKNVYTLFEIQNVENSEKFYKYMAKHYKNS-PCDDA-FECHEQGIKTARQFAEFMKIKL
YP_009037398.1       GIKNVYTLFEIQNVENSEKFYKYMAKHYKNS-PCDDA-FECHEQGIKTARQFAEFMKIKL
YP_010100079.1       GIKNVYTLFEIQNVENSEKFYKYMAKHYKNS-PCDDA-FECHEQGIKTARQFAEFMKIKL
WPK34318.1           GIKNVYTLFEIQNVENSEKFYKYMAKHYKNS-PCDDA-FECHEQGIKTARQFAEFMKIKL
```

```
YP_010088390.1      GIKNVYTLFEIQNVENSEKFYKYMAKHYKNS-PCDDA-FECHEQGIKTARQFAEFMKIKL
UOL50774.1          GIKNVYTLFEIQNVENSEKFYKYMAKHYKNS-PCDDA-FECHEQGIKTARXFAEFMKIKL
YP_010076184.1      GIKNVYTLFEIQNVENSEKFYKYMAKHYKNS-PCDDA-FECHEKGIKTARQFAEFMKIKL
QHR68209.1          GIKNVYTLFEIQNVENSEKFYKYMAKHYKNS-PCDDA-FECYEQGIKTARQFAEFMKIKL
QHR72707.1          GIKNVYTLFEIQNVENSEKFYKYMAKHYKNS-PCDDA-FECHEQGIKTARQFAEFMKIKL
YP_009225157.1      GIKNVYTLFEIQNVENSEKFYKYMAKHYKNS-PCDDA-FECHEQGIKTARQFAEFMKIKL
CAH1486843.1        GIKNVYTLFEIQNVENSEKFYKYMAKHYKNS-PCDDA-FECHEQGIKTARQFAEFMKIKL
YP_010068772.1      GIKNVYTLFEIQNVENSEKFYKYMAKHYKNS-PCDDA-FECHEQGIKTARQFAEFMKIKL
YP_009608297.1      GIKNVYTLFEIQNVENSEKFYKYMAKHYKNS-PCDDA-FECHEQGIKTARQFAEFMKIKL
YP_010246930.1      GIKNVYTLFEIQNVENSEKFYKYMAKHYKNS-PCDDA-FECHEQGIKTARQFAEFMKIKL
ULA52136.1          GIKNVYTLFEIQNVENSEKFYKYMVKHYKNS-PCDDA-FECHEQGIKTARQFAEFMKIKL
                    .   :*      .   *  :  :    :

QPI17802.1          -------------
QFR55726.1          EDAKF--------
YP_009607330.1      -------------
URQ04387.1          -------------
YP_009194379.1      -------------
YP_009607052.1      -------------
YP_003579989.1      -------------
YP_007348772.1      -------------
UIW11501.1          -------------
DAG47327.1          -------------
YP_009194955.1      -------------
YP_003858432.1      -------------
YP_009097745.1      -------------
YP_006986407.1      -------------
QUL77064.1          -------------
UIW12440.1          -------------
QJI53183.1          LTK----------
YP_009205796.1      EIQ----------
UPW37817.1          EIQ----------
CAI9865938.1        EIQ----------
YP_001595227.1      EIQ----------
WPJ21437.1          EVQ----------
QAY00131.1          EIQ----------
YP_003734244.1      EIQ----------
QBP35713.1          EVQ----------
YP_009126310.1      EEHEI--------
YP_007501144.1      EEHEI--------
YP_009286464.1      EENEI--------
YP_009615586.1      EENEI--------
QQG32244.1          EDNEI--------
WKV23456.1          KHEI---------
YP_009146546.1      KHEI---------
YP_009203826.1      KHEI---------
YP_004010271.1      KPNPSLQSK----
YP_006488804.1      KPKKSVQIIKTSL
YP_009882281.1      KPKSSLQPSKRSI
UQS93713.1          KAKK---------
YP_009880905.1      KAKK---------
YP_009881568.1      KAKK---------
YP_009889763.1      KAKK---------
QKN88074.1          KAKK---------
YP_009886406.1      KAKK---------
YP_004300701.1      KEKDNK-------
YP_004009500.1      EKK----------
YP_010093716.1      VHDE---------
QPB12267.1          EINEALIGNMENT
YP_009211536.1      NEDES--------
QMP18845.1          EDEIR--------
YP_004063793.1      EDEIR--------
QQG30800.1          EDEIR--------
QUL77647.1          EDEI---------
YP_009209851.1      EDEIR--------
UHS65324.1          EDEI---------
QIN95484.1          EDEI---------
YP_003934732.1      EDEI---------
QWQ55747.1          EDEI---------
WCZ66167.1          ENELQ--------
YP_010090811.1      ENEI---------
YP_010092259.1      DNEIQ--------
UYM28759.1          DNEIQ--------
YP_009609435.1      DNEIQ--------
YP_009883816.1      EHDGI--------
YP_009030155.1      EHDGI--------
UES35696.1          ENEI---------
YP_009005374.1      ENEI---------
YP_004009968.1      ENEI---------
WJZ27985.1          EK-----------
QPI14021.1          ES-----------
YP_010090638.1      DGDY---------
YP_009850289.1      DHEVQ--------
YP_009836720.1      DHEVQ--------
WKW88073.1          DHEVQ--------
QPB08927.1          DNEVQ--------
QEG11753.1          DNEVQ--------
YP_009190675.1      DNEVQ--------
WPH67859.1          DNEVQ--------
```

```
YP_009289456.1      DNEVQ--------
UGO47466.1          DNEVQ--------
QPX73789.1          DNEVQ--------
UNY41189.1          DNEVQ--------
UGO48449.1          DNEVQ--------
UMM76594.1          DNEIQ--------
UGO49742.1          DNEIQ--------
YP_010089418.1      DNEVQ--------
YP_009288785.1      DNEVQ--------
UOK17715.1          DNEVQ--------
UGO53259.1          DNEVQ--------
WAB00422.1          DNEVQ--------
QYC52795.1          DNEVQ--------
QOI66588.1          DPEHT--------
YP_010095043.1      DPDNS--------
YP_010089700.1      DDENK--------
YP_007235940.1      DNENK--------
YP_009200371.1      DNENH--------
AIT75019.1          -------------
YP_010071311.1      ETE----------
QXV84950.1          ETE----------
WBY53229.1          -------------
QOQ37471.1          ETE----------
YP_010070761.1      ETE----------
WAE77443.1          ETE----------
WBF79919.1          ETE----------
ULA51879.1          EIE----------
WAX13030.1          ETE----------
YP_010072394.1      ETE----------
YP_004415003.1      ETE----------
YP_010071150.1      ETE----------
YP_010091143.1      ETE----------
YP_010073757.1      ETE----------
YP_010070485.1      ETE----------
YP_010077370.1      ETE----------
UDY80501.1          ETE----------
QWV60450.1          ETE----------
YP_007004487.1      ETE----------
YP_009153709.1      ETE----------
UIU27783.1          ETE----------
URY11966.1          ETE----------
YP_002854065.1      ETE----------
YP_009614761.1      ETE----------
YP_010069670.1      ETE----------
YP_009110927.1      ETE----------
QXN75810.1          ETE----------
YP_006986660.1      ETE----------
YP_009277482.1      ETE----------
YP_009210294.1      ETE----------
YP_002854444.1      ETE----------
QXV79213.1          ETE----------
YP_010106139.1      ETE----------
QOC55302.1          ETE----------
URY14984.1          ETE----------
WJJ57515.1          ETE----------
NP_049717.1         ETE----------
QBO63621.1          ETE----------
UPW39297.1          NDSF---------
QEG06222.1          RAYIYLIY-----
QBZ70948.1          RAYIYLIY-----
YP_009056689.1      EPTSI--------
QSL99198.1          EPTSI--------
YP_009100645.1      EPTSI--------
QBP05720.1          EPTSI--------
AYR04018.1          ESTSI--------
YP_009111273.1      EPTSI--------
NP_861799.1         EPTSI--------
YP_010088590.1      EPTSI--------
WPK33788.1          EPTSI--------
QVW29258.1          EPKSI--------
QHR76563.1          EPTSI--------
WPK34047.1          EPTSI--------
QXV77046.1          EPTSI--------
UUB18216.1          EPTSI--------
YP_010096345.1      EPTSI--------
AWM11858.1          EPTSI--------
WBF80716.1          EPTSI--------
YP_009592859.1      EPTSI--------
YP_009037398.1      EPTSI--------
YP_010100079.1      EPTSI--------
WPK34318.1          EPTSI--------
YP_010088390.1      EPTSI--------
UOL50774.1          EPTSI--------
YP_010076184.1      EPTSI--------
QHR68209.1          EPTSI--------
QHR72707.1          EPTSI--------
YP_009225157.1      EPTSI--------
CAH1486843.1        EPTSI--------
YP_010068772.1      EPTSI--------
```

```
YP_009608297.1      EPTSI--------
YP_010246930.1      EPTSI--------
ULA52136.1          EPTSI--------
```

Supplementary Table S2. Names of the phages used for the alignment.

**GenBank accession numbers (ordered as in the alignment)**	**Phage names (E. coli phage T4 highlighted in yellow)**
QPI17802.1	*Pectobacterium* phage POP1
QFR55726.1	*Yersinia* phage JC221
YP_009607330.1	*Klebsiella* phage Miro
URQ04387.1	*Klebsiella* phage BL02
YP_009194379.1	*Klebsiella* phage Matisse
YP_009607052.1	*Enterobacter* phage phiEap3
YP_003579989.1	*Klebsiella* phage KP15
YP_007348772.1	*Klebsiella* phage KP27
UIW11501.1	*Enterobacter* phage ENC7
DAG47327.1	*Caudoviricetes* sp.
YP_009194955.1	*Citrobacter* phage Margaery
YP_003858432.1	*Escherichia* phage RB16
YP_009097745.1	*Citrobacter* phage Miller
YP_006986407.1	*Cronobacter* phage vB_CsaM_GAP161
QUL77064.1	*Escherichia* phage UPEC03
UIW12440.1	*Enterobacter* phage ENC20
QJI53183.1	*Enterobacter* phage EBPL
YP_009205796.1	*Escherichia* phage vb_EcoMVR5
UPW37817.1	*Escherichia* phage vB_EcoM_ESCO10
CAI9865938.1	*Escherichia* phage UP19
YP_001595227.1	*Escherichia* phage JS98
WPJ21437.1	*Salmonella* phage vB_SalD_ABTNLS3
QAY00131.1	*Escherichia* phage EcWhh1
YP_003734244.1	*Escherichia* phage IME08
QBP35713.1	*Escherichia* Phage NCG
YP_009126310.1	*Salmonella* phage STP4a
YP_007501144.1	*Salmonella* phage vB_SenMS16
YP_009286464.1	*Salmonella* phage vB_SnwM_CGG41
YP_009615586.1	*Salmonella* phage Melville
QQG32244.1	*Citrobacter* phage CkP1
WKV23456.1	*Salmonella* phage SEA1
YP_009146546.1	*Citrobacter* phage Moon
YP_009203826.1	*Citrobacter* phage Merlin
YP_004010271.1	*Acinetobacter* phage Acj9
YP_006488804.1	*Acinetobacter* phage ZZ1
YP_009882281.1	*Acinetobacter* phage AbTZA1
UQS93713.1	*Acinetobacter* phage AC4
YP_009880905.1	*Acinetobacter* phage vB_ApiM_fHyAci03
YP_009881568.1	*Acinetobacter* phage KARL1
YP_009889763.1	*Acinetobacter* phage AM101
QKN88074.1	*Acinetobacter* phage Abraxas
YP_009886406.1	*Acinetobacter* phage vB_AbaM_Apostate
YP_004300701.1	*Acinetobacter* phage 133
YP_004009500.1	*Acinetobacter* phage Ac42
YP_010093716.1	*Proteus* phage phiP43
QPB12267.1	*Providencia* phage PSTCR6
YP_009211536.1	*Pectobacterium* bacteriophage PM2
QMP18845.1	*Escherichia* phage CJ20
YP_004063793.1	*Escherichia* phage vB_EcoM_VR7
QQG30800.1	*Escherichia* phage UPEC01
QUL77647.1	*Escherichia* phage UPEC07
YP_009209851.1	*Escherichia* phage vB_EcoM_VR25
UHS65324.1	*Escherichia* phage P896
QIN95484.1	*Escherichia* phage MN01
YP_003934732.1	*Shigella* phage SP18
QWQ55747.1	*Escherichia* phage P479
WCZ66167.1	*Yersinia* phage MHG19
YP_010090811.1	*Escherichia* phage EcS1
YP_010092259.1	*Serratia* phage X20
UYM28759.1	*Serratia* phage vB_SspM_LC53
YP_009609435.1	*Serratia* phage CHI14
YP_009883816.1	*Serratia* phage Muldoon
YP_009030155.1	*Serratia* phage PS2
UES35696.1	*Enterobacter* phage KKP_3262
YP_009005374.1	*Enterobacter* phage PG7

YP_004009968.1	*Enterobacter* phage CC31
WJZ27985.1	*Serratia* phage 92A1
QPI14021.1	*Serratia* phage 4S
YP_010090638.1	*Pseudomonas* phage PspYZU05
YP_009850289.1	*Klebsiella* phage Marfa
YP_009836720.1	*Klebsiella* phage vB_Kpn_F48
WKW88073.1	*Klebsiella* phage pzkkv8
QPB08927.1	*Klebsiella* phage Metamorpho
QEG11753.1	*Klebsiella* phage KPN6
YP_009190675.1	*Klebsiella* phage JD18
WPH67859.1	*Klebsiella* phage ValerieMcCarty01
YP_009289456.1	*Klebsiella* phage PKO111
UGO47466.1	*Klebsiella* phage vB_KaeM_LilPanda
QPX73789.1	*Klebsiella* phage vB_KpnM_BovinicusUrsus
UNY41189.1	*Klebsiella* phage KP185
UGO48449.1	*Klebsiella* phage vB_KaeM_Boboto
UMM76594.1	*Klebsiella* phage UTIK1
UGO49742.1	*Klebsiella* phage vB_KaeM_Merci
YP_010089418.1	*Klebsiella* phage KPV15
YP_009288785.1	*Klebsiella* phage vB_KpnM_KpV477
UOK17715.1	*Klebsiella* phage KP1079
UGO53259.1	*Klebsiella* phage vB_KaeM_Nispero
WAB00422.1	*Klebsiella* phage JY1
QYC52795.1	*Klebsiella* phage vB_KpnM_TU02
QOI66588.1	*Erwinia* phage FBB1
YP_010095043.1	*Erwinia* phage Cronus
YP_010089700.1	*Yersinia* phage fHeYen91
YP_007235940.1	*Yersinia* phage phiR1RT
YP_009200371.1	*Yersinia* phage vB_YenM_TG1
AIT75019.1	*Enterobacteria* phage RB55
YP_010071311.1	*Escherichia* phage vB_EcoM_KAW1E185
QXV84950.1	*Escherichia* phage TadeuszReichstein
WBY53229.1	*Escherichia* phage REP4
QOQ37471.1	*Escherichia* phage vB_EcoM_WL3
YP_010070761.1	*Escherichia* phage vB_EcoM_G906
WAE77443.1	*Escherichia* phage ph0021
WBF79919.1	*Escherichia* phage vB_Eco_F25
ULA51879.1	*Escherichia* phage vB_EcoMRPN226
WAX13030.1	*Escherichia* phage ECO07P2
YP_010072394.1	*Escherichia* phage vB_EcoM_OE5505
YP_004415003.1	*Shigella* phage Shfl2
YP_010071150.1	*Escherichia* phage vB_EcoM_IME537
YP_010091143.1	*Yersinia* phage fPS90
YP_010073757.1	*Escherichia* phage T2
YP_010070485.1	*Escherichia* phage vB_EcoM_G4507
YP_010077370.1	*Yersinia* phage PYPS2T
UDY80501.1	*Shigella* phage CT01
QWV60450.1	*Escherichia* phage W143
YP_007004487.1	*Escherichia* phage ime09
YP_009153709.1	*Yersinia* phage PST
UIU27783.1	*Escherichia* phage vB_EcoM_SA21RB
URY11966.1	*Shigella* phage ESh17
YP_002854065.1	*Enterobacteria* phage RB51
YP_009614761.1	*Shigella* phage Sf22
YP_010069670.1	*Escherichia* phage vB_EcoMG28
YP_009110927.1	*Shigella* phage pSs1
QXN75810.1	*Escherichia* phage BF15
YP_006986660.1	*Escherichia* phage vB_EcoM_ACGC40
YP_009277482.1	*Shigella* phage SHFML11
YP_009210294.1	*Escherichia* phage slur02
YP_002854444.1	*Escherichia* phage RB14
QXV79213.1	*Escherichia* phage FriedrichZschokke
YP_010106139.1	Phage NBEco003 (host not indicated)
QOC55302.1	*Escherichia* phage JEP6
URY14984.1	*Shigella* phage ESh31
WJJ57515.1	*Escherichia* phage 4E10
NP_049717.1	*Escherichia* phage T4
QBO63621.1	*Escherichia* phage vB_EcoM_G10400
UPW39297.1	*Escherichia* phage vB_EcoM_ESCO47
QEG06222.1	*Shigella* phage JK42
QBZ70948.1	*Shigella* phage SSE1
YP_009056689.1	*Escherichia* phage vB_EcoM_PhAPEC2

QSL99198.1	*Escherichia* phage PTK
YP_009100645.1	*Shigella* phage Shf125875
QBP05720.1	*Escherichia* phage PHB12
AYR04018.1	*Escherichia* phage OLB35
YP_009111273.1	*Escherichia* phage Av 5
NP_861799.1	*Escherichia* phage RB69
YP_010088590.1	*Escherichia* phage phiE142
WPK33788.1	*Escherichia* phage AV109
QVW29258.1	*Escherichia* phage vB_EcoM_SQ17
QHR76563.1	*Escherichia* phage moskry
WPK34047.1	*Escherichia* phage AV110
QXV77046.1	*Escherichia* phage ChristianSchoenbein
UUB18216.1	*Escherichia* phage ST2
YP_010096345.1	*Escherichia* phage SF
AWM11858.1	*Escherichia* phage vB_EcoM_NBG1
WBF80716.1	*Escherichia* phage vB_Eco_F31
YP_009592859.1	*Escherichia* coli O157 typing phage 3
YP_009037398.1	*Escherichia* phage vB_EcoM_JS09
YP_010100079.1	*Escherichia* phage p000v
WPK34318.1	*Escherichia* phage AV111
YP_010088390.1	*Enterobacteria* phage ATK47
UOL50774.1	*Escherichia* phage vB_EcoM_SCS57
YP_010076184.1	*Shigella* phage JK45
QHR68209.1	*Escherichia* phage moha
QHR72707.1	*Escherichia* phage mobillu
YP_009225157.1	*Escherichia* phage APCEc01
CAH1486843.1	*Escherichia* phage UGJNEcP1
YP_010068772.1	*Escherichia* phage F2
YP_009608297.1	*Escherichia* phage ST0
YP_010246930.1	*Escherichia* phage HX01
ULA52136.1	*Escherichia* phage vB_EcoM RPN187

Supplementary Figure S1. Effect of point mutations in RI and T on LIN.

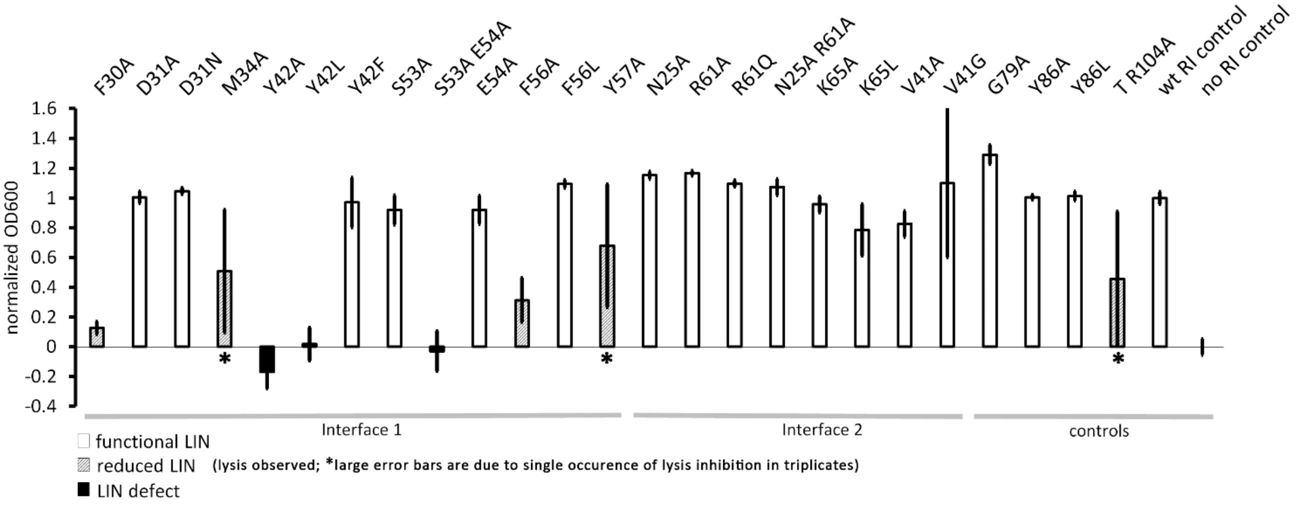

Supplement: Supplementary file 1 [file Data_Sheet_1.PDF]
